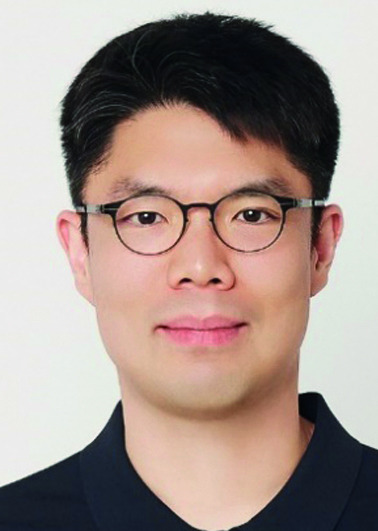# Valorisation of Greenhouse Gas for Commodity Chemical Production–Nitrous Oxide as Oxidising Agent for Heterogeneous Catalysis

**DOI:** 10.1002/cssc.202402728

**Published:** 2025-04-03

**Authors:** Seonho Lee, Seong‐Jik Park, Jechan Lee

**Affiliations:** ^1^ Department of Global Smart City Sungkyunkwan University Suwon 16419 Republic of Korea; ^2^ Department of Integrated System Engineering & Department of Bioresources and Rural System Engineering Hankyong National University Anseong 17579 Republic of Korea; ^3^ School of Civil Architectural Engineering and Landscape Architecture Sungkyunkwan University Suwon 16419 Republic of Korea

**Keywords:** N_2_O utilisation, N_2_O oxidant, greenhouse gas reduction, catalyst modification, chemical conversion

## Abstract

Nitrous oxide (N_2_O) has shown application potential as an oxidant to directly convert substances such as methane, ethane, and benzene into useful substances such as methanol, styrene, phenols, and carbonyl compounds. It decomposes greenhouse gases and simultaneously produces highly applicable substances. Research have been performed to improve the conversion of N_2_O and reactants in this process and to increase the selectivity of useful chemicals. Catalyst modifications have been tried to favour the production of reactive oxygen species and prevent further oxidation for better performances. This review paper focuses on various methods to increase the efficiency of heterogeneous catalytic N_2_O‐assisted selective oxidation within the last five years, particularly on catalyst improvements. Results are categorised based on targeted products (oxygenates, hydrocarbons and ring compounds), revealing the approaches and achievements of each researcher. The catalytic performance can be optimised by changing the phase, surface area and acidity of the catalyst through metal loading and changes in manufacturing methods. To increase the practicality of this technology, additional research needs to be performed.

## Graphical abstract

Heterogeneous catalysts can enhance the selectivity of oxidation reactions using N_2_O as oxidizing Agent with proper modification of catalyst phase, surface area, and acidity. N₂O activation leads to generating reactive oxygen species, facilitating selective oxidation and dehydrogenation to produce various products such as oxygenates, hydrocarbons, and ring compounds, contributing to sustainable production of value‐added chemicals.

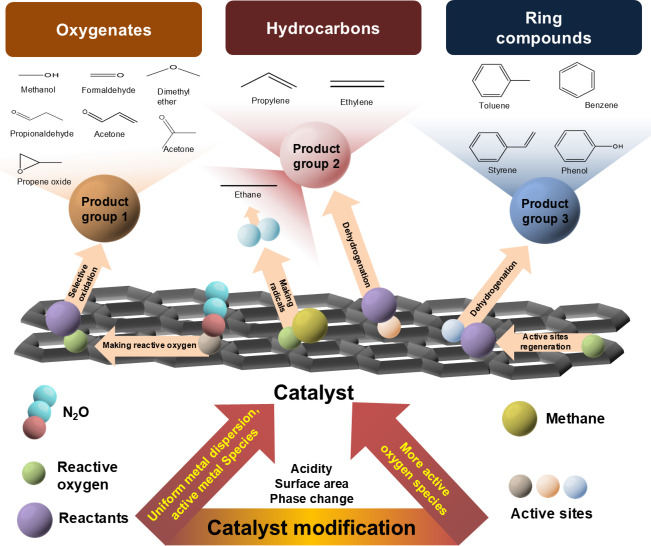

## 1 Introduction

Nitrous oxide (dinitrogen oxide; N_2_O) is a colourless non‐flammable gas having sweet scent and taste at room temperature. At high temperatures, N_2_O serves as a strong oxidiser and promoter of hydrocarbon and hydrogen combustion.^[1]^ Notably, N_2_O is a potent greenhouse gas whose global warming potential is 265 times greater than that of carbon oxide (CO_2_) over a 100‐year timescale.^[2]^ In addition, N_2_O is a dominant ozone‐depleting substance in the 21st century.^[3]^ Although the specific sources and magnitudes of N_2_O are contested, agricultural soils are considered as a major source of N_2_O emissions because of the widespread use of synthetic nitrogen fertilisers and livestock manure.^[4]^ N_2_O is produced as a by‐product of abiotic redox reactions and biological processes (*e. g*., nitrification, denitrification and co‐denitrification) occurring in soil.^[5]^ Global N_2_O emissions are expected to increase because of the accelerated nitrification and denitrification in soil due to global warming and growing fertiliser industry.^[6]^

Several methods have been proposed to reduce N_2_O emissions from agricultural activities, including soil amendment, controlled biological processes and adsorption using biochar.[^7^, ^8^] However, the effectiveness of such methods is highly associated with the physicochemical properties of soil, rendering the performance of reducing N_2_O emissions inconsistent.^[9]^ Moreover, biochar may contain species substances that inhibit microbial activity (*e. g*., heavy metals, polycyclic aromatic hydrocarbons, volatile organic compounds, polychlorinated dibenzo‐p‐dioxins and furans)^[10]^ and cause air, water and soil pollution.^[11]^

In addition to agricultural sector, considerable amounts of N_2_O are emitted from industrial sites, particularly in nitric acid and adipic acid manufacturing, biomass combustion and wastewater treatment.^[12]^ Therefore, various methods have been suggested, including catalytic decomposition, selective adsorption and high‐efficiency combustion, to reduce N_2_O emissions from the aforementioned sources.^[13]^ Furthermore, several attempts have been made for using N_2_O as a chemical agent (*e. g*., terminal oxidant and oxygen donor).^[14,15][15]^Considering the energetic favourability of oxygen dissociation from N_2_O and tendency of N_2_O to avoid overoxidation, N_2_O is regarded as an effective reagent for oxidation reactions, such as the epoxidation of alkenes, hydroxylation of arenes and oxidation of alcohols.[^16^, ^17^, ^18^, ^19^]

Compared with conventional N_2_O mitigation approaches, using N_2_O as an oxidant is an attractive strategy for achieving N_2_O emission reduction and producing useful commodity chemicals. Research on utilizing N₂O as an oxidizing agent to enhance the yield of commercially valuable chemicals is a worthwhile pursuit. This review paper covers gas phase catalytic oxidation assisted by N_2_O using heterogeneous solid catalysts. As review papers summarising and focusing on homogeneous process^[20]^ or non‐catalytic^[21]^ are relatively recent, we have not discussed that in this paper. Gas phase reactions over heterogeneous catalysts have the benefits of easy product separation, simple management.^[22]^ Despite continuous study, there are currently no review papers that comprehensively cover the latest research trends in this area, to the best of the author‘s knowledge. Therefore, this review aims to provide the recent research achievements in improving the yield of highly applicable chemicals using N_2_O as an oxidation agent over the last five years. In addition to the production of phenol from benzene and methanol from methane, which were relatively early studies, it includes several recent studies. This paper categorises the products that researchers have targeted in N_2_O‐assisted oxidation reactions. The target products are divided into three main categories—oxygenates, hydrocarbons and ring compounds—and presented in separate sections. This paper shows the researchers attempts in designing catalysts and experimental conditions to increase the efficiency of reactions using N_2_O as an oxidant. This review will help researchers in related fields to make more advanced research attempts based on previous results.

## 2 Reactants and Products from N_2_O‐Assisted Chemical Process

**Figure 1:**
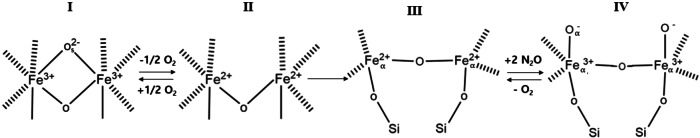
Alpha site formation mechanism. Reproduced from Starokon et al.^[24]^ with permission from Springer Nature.

Candidate substances that can be used as oxidants in chemical processes (*e. g*., O_2_, N_2_O, H_2_O, and CO_2_) have different coordination and binding energies; therefore, they have different interaction capabilities and tendencies with catalysts.^[23]^ Initially, the main focus was on using N_2_O as an oxidant for the selective oxidation of methane. It has been proven that zeolites containing iron have an active site called the alpha site that only functions under N_2_O. Figure 1 shows the mechanism of generating alpha site. In Fe‐containing zeolites, such as FeZSM‐5, form (I) arises due to Fe migration and external Fe introduction. Upon desorption of oxygen at high temperatures, it exists in the same form as (II). However, this (II) form is reversible. It reverts to the (I) form upon cooling. To create an alpha site, reduced Fe^2+^ must lose its ability to react with O_2_. Treatments such as steam activation or modification of the zeolite structure can cause irreversible desorption of oxygen and create alpha sites. The stabilization of the alpha site occurs in the (III) form. In this form, the iron atom can be easily oxidized to form (IV) by N_2_O, producing adsorbed oxygen species. The reversible redox transition between (III) and (IV) allows for the selective transfer of oxygen atoms from N_2_O to hydrocarbon molecules.^[24]^

Research on selective oxidation using the unique properties of N_2_O has been applied to the process of oxidizing benzene to phenol,^[25]^ and has been expanded to a wider range of reactions (*e. g*., converting propane to propylene). Recently, N_2_O has been utilized as an oxidant in oxidative dehydrogenation reactions (production of styrene from ethylbenzene., etc). Research results have shown that N_2_O achieves higher selectivity than O_2_, CO_2_, and other oxidants. N_2_O can mitigate the limitations of other oxidant candidates such as sulfur dioxide (SO_2_), carbon oxide (CO_2_) and oxygen (O_2_). O_2_ can cause peroxidation, CO_2_ can cause excessive coking and SO_2_ has issues such as creating corrosive materials. This field of study combined with the benefits of reducing environmental pollutants that contribute to the greenhouse effect has emerged as an interesting research topic.^[26]^ Many transition metal species have been studied that act as active centers for the decomposition and activation of N_2_O. Among the low‐cost transition metals, the most active are Fe, Co, Ni, and Cu, in that order. Some noble metals such as Ru and Rh show even higher N_2_O degradation capabilities, but they are easily poisoned by NO, O_2_, and H_2_O and are not suitable for large‐scale applications due to their high cost. Recent research continues to focus on catalyst design to overcome the limitations of each transition metal and facilitate the selective delivery of oxygen species from N_2_O decomposition to the reactants.^[27]^

The reactants and widely applicable chemicals that could potentially be obtained from N_2_O‐using processes referred to in this paper are shown in Table 1. Table 1 demonstrates that the products from N₂O‐assisted oxidation processes are high‐demand chemicals. This implies that N₂O utilization offers not only environmental benefits but also economic potential. In summary, the development and industrialization of N₂O utilization technology is essential. A detailed description of the research cases mentioned in Table 1 is presented in Sections 3–5.

**Table 1:** Projected market value of products potentially obtained from N₂O‐assisted processes.

Reactant	Products	Products utilisation	Market value (USD billion)	Ref.
Methane	Methanol	Chemical industry, fuel cell, construction, automotive, electronics, appliances, paints, pharmacy, and plastics	46.32 in 2032	[28,29]
	Formaldehyde	Adhesive, chemical industry, resin, insulation material, etc	44.99 in 2031	[30,31]
	DME	Fuel, refrigerant, reaction solvent, pesticide, fuel cell applications, chemical intermediate (ethane, propane, methyl acetate, dimethyl sulphate, etc.)	10.02 in 2030	[32,33]
	Ethylene	Chemical industry, plastic manufacture, etc.	246.86 in 2032	[34,35]
	Ethane	Ethylene, ethanol production, etc.	17.8 by 2030	[36,37]
Propene	Propene oxide	Chemical industry, solvent, etc.	25,356.9 million by 2032	[38,39]
Ethane	Ethylene	Chemical industry, plastic manufacture, etc.	246.86 in 2032	[34,40]
Ethylbenzene	Styrene	Chemical industry, packaging, etc.	97.31 in 2032	[41,42]
Benzene	Phenol	Resin industry (adhesive, paints, coating, insulating foams), chemical industry, epoxies, etc.	28.99 in 2030	[43,44]

## 3 Oxygenate Products

In this section, the production of various oxygenated compounds (*e. g*., methanol, dimethyl ether (DME), acetone (ACT) and carbonyl products such as formaldehyde) using N_2_O as an oxidising agent is discussed. Methane is primarily used to produce high‐value‐added substances such as methanol, formaldehyde and DME. Methane is a relatively cheap, abundant natural resource and an important alternative to crucial oil to produce high‐value‐added substances. The main product of the partial oxidation process with N_2_O and methane is methanol.

Iron‐modified ZSM‐5 has been attracting attention as an effective catalyst system in the nitrogen dioxide‐mediated selective oxidation reaction of methane. Penov et al. discovered that N_2_O decomposition in iron complexes stabilized in a ZSM‐5 matrix produces a specific form of atomic oxygen, namely alpha oxygen, at room temperature.^[45]^ Research to improve the activity of catalysts in the N_2_O‐mediated selective oxidation of methane has continued. Zhao et al. incorporated Fe into different frameworks (ZSM‐5, Beta, FER) and analyzed the characteristics of the Fe species in the catalysts, N_2_O conversion and product selectivity.^[46]^ Fe‐FER showed superior performance compared with Fe‐ZSM‐5 and Fe‐BETA. Fe‐FER exhibited higher methane conversion and N_2_O conversion than the other catalysts. The methane conversion of Fe‐FER was 2.4 %, which was 1.7 times that of Fe‐ZSM‐5 and 1.8 times that of Fe ‐BETA. The N_2_O conversion of Fe‐FER was 20 %, which was 2.8 times that of Fe‐ZSM‐5 and 2.2 times that of Fe‐BETA. Fe‐FER also had a greater selectivity of reactants than other catalysts because of the following reasons: First, the Fe‐FER catalyst had additional active sites and, thus, more specific oxygen. Fe‐FER has the largest number of framework Al atoms, which was beneficial for stabilizing Fe species and creating active sites required for N_2_O conversion. Second, the methanol intermediate species migrated more efficiently. Fe‐FER has a large amount of silanol‐bonded methoxy groups. In other words, methane molecules were converted to methoxy by reactive oxygen species, and movement and adsorption to silanol groups occur rapidly. Conversely, Fe‐ZSM‐5 and Fe‐BETA catalysts tended to accumulate methoxy groups at the Fe site rather than migrating to silicon species. Therefore, the active sites were better vacated, resulting in a promoted reaction and higher methanol selectivity (21 % for Fe‐FER, 4.7 % for Fe‐BETA and 3.1 % for Fe‐ZSM‐5). This reaction led to the relatively active dehydration of methanol, resulting in a higher selectivity to DME. In addition, Fe‐FER had the highest number of Brønsted acid sites where methanol dehydration occurs (Table 2, entry 3). The Brønsted acid site concentrations were 1.26, 0.97, and 0.86 mmol/g in the order of Fe‐FER, Fe‐BETA, and Fe‐ZSM‐5, respectively. This catalyzed methanol dehydration, leading to the production of DME. Therefore, only Fe‐FER had a high DME selectivity of 20.6 %. Fe‐ZSM‐5 was noteworthy for its high ethylene selectivity of 31 %. It was attributed to the appropriate pore size and low concentration of Lewis sites (Table 2, entry 1).^[46]^

**Table 2:** Characteristics of catalyst for conversion to oxygenates.

Entry	Catalyst	Method	Metal loading	Calcination condition	Sieve(μm)	Concentration of acid sites(mmol/g)	Surface area (m^2^/g)	Micropore area (m^2^/g)	Mesopore area (m^2^/g)	Micropore volume (cm^3^/g)	Total pore volume (cm^3^/g)	Average pore diameter (nm)	Ref.	Entry	Catalyst	Method	Metal loading	Calcination condition
			Metal	Ratio (wt %)	Dispersion (%)	pH			Brønsted	Lewis	Total							
1	Fe‐ZSM‐5	Incipient wetness impregnation	Iron (III) nitrate nonahydrate	Fe (2.3)	12.1	‐	550 °C; 300 min; 3 °C/min	250–425	0.86	0.12	‐	‐	476	‐	0.11	‐	‐	[46]
2	Fe‐BETA	Incipient wetness impregnation	Iron (III) nitrate nonahydrate	Fe (2.3)	12.8	‐	550 °C; 300 min; 4 °C/min	250–425	0.97	0.38	‐	‐	929	‐	0.17	‐	‐	
3	Fe‐FER	Incipient wetness impregnation	Iron (III) nitrate nonahydrate	Fe (2.2)	14.6	‐	550 °C; 300 min; 5 °C/min	250–425	1.26	0.3	‐	‐	438	‐	0.13	‐	‐	
4	Fe‐FER‐0.2	Solid‐state ion exchange method and desilication with NaOH solution	Iron (III) chloride hexahydrate	Fe (0.53)	‐	‐	500–550 °C; 180–300 min	250–425					226	87.6				[50]
5	Fe‐FER‐SSIE	Solid‐state ion exchange	Iron (III) chloride hexahydrate	Fe (0.49)	‐	‐	500–550 °C; 180–300 min; 3 °C/min					‐	253.8		0.13	‐	‐	[49]
6	Fe‐FER‐IE	Liquid ion exchange	Iron (III) chloride hexahydrate	Fe (0.53)	‐	‐	550 °C;300 h; 3 °C/min					‐	253.4		0.14	‐	‐	
7	FeZSM‐F	Incipient wetness impregnation	Iron (III) nitrate nonahydrate	Fe (1)	‐	‐	550–900 °C; 60 min	20–40 mesh	‐	‐	‐	268	‐	‐	0.1	‐	‐	[51]
8	FeZSM‐E	Incipient wetness impregnation	Iron (III) nitrate nonahydrate	Fe (1)	‐	‐	550–900 °C; 60 min	20–40 mesh	‐	‐	‐	278	‐	‐	0.104	‐	‐	
9	FePO_4_‐tdm	Ammonia gel	Iron (III) nitrate nonahydrate	Fe	27.8	‐	500 °C; 240 min	‐	‐	‐	‐	19	‐	‐	‐	0.021	28	[52]
10	Cu‐Fe/‐Al_2_O_3_(COP)	Co‐precipitation	Metal nitrates and r‐Al_2_O_3_	Cu (5), Fe (5)	49	‐	400 °C; 240 min	‐	‐	‐	‐	112	‐	‐	‐	0.19	12	[23]
11	Cu‐Fe/‐Al_2_O_3_(hydrothermal)	Hydrothermal	Copper (II) nitrate hydrate and Iron (III) nitrate nonahydrate	Cu (5), Fe (5)	63	‐	400 °C; 240 min	‐	‐	‐	‐	153	‐	‐	‐	0.27	8	
12	5Cu/AEI(Na)‐750	Using organic structure‐directing agent and ion exchange	Copper (II) nitrate and Sodium hydroxide	Cu (0.78)	‐	‐	550 °C; 300 min & 750 °C; 600 min	500–1000	0.43	0.56	1.11	437	‐	‐	0.2	‐	‐	[55]
13	5Cu/AEI(Na free)‐750	Using organic structure‐directing agent and ion exchange	Copper (II) nitrate	Cu (0.89)	‐	‐	550 °C; 300 min & 750 °C; 600 min	500–1000	0.2	0.32	0.93	479	‐	‐	0.23	‐	‐	
14	VS1‐SBA‐3	One‐pot hydrothermal procedure	Vanadium (IV) oxide sulfate hydrate	Vanadium (0.48)	‐	<1	500 °C; 480 min	‐	‐	‐	0.036	1411	‐	‐	‐	0.74	2.09	[61]
15	VM1‐SBA‐3	One‐pot hydrothermal procedure	Ammonium metavanadate	Vanadium (0.26)	‐	<1	500 °C; 480 mn	‐	‐	‐	0.023	1370	‐	‐	‐	0.73	2.1	

Controlling migration–recombination mechanism is important factor to improving product selectivity in selective oxidation process. Oxygen molecules have been found to be generated by migration–recombination mechanisms,^[47]^ with the decomposition of surface oxygen species releasing oxygen molecules.^[48]^ It oxidize products (*e. g*., methanol, formaldehyde) to carbon oxide resulting in a decrease in selectivity.^[49]^

**Figure 2:**
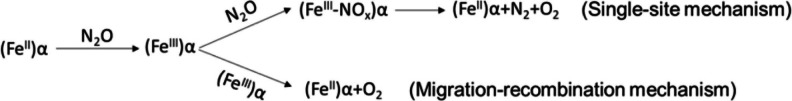
Pathway of migration‐recombination mechanism and single‐site mechanism. Reprinted from Zhao et al.,^[50]^ Copyright (2022), with permission from Elsevier.

To minimize the decomposition of surface oxygen species through migration–recombination **(**Figure 2 **)**, The desilication of Fe‐FER by treatment with NaOH solutions (0.1, 0.2, and 0.3 M) prior to the loading of iron ion was performed.^[50]^ Alkaline treatment removed the binding sites of catalytically active iron species, which inhibited their catalytic activity for N_2_O decomposition. However, the methane conversion rate of Fe‐FER‐0.1 M and Fe‐FER‐0.2 M was relatively high (about 2.8 %). This result was due to the compensation for the weakening of N_2_O conversion. First, the high utilisation efficiency of N_2_O was due to the decrease in the migration–recombination reaction of active oxygen species caused by alkali treatment. Second, the utilisation efficiency of reactive oxygen species was improved. The self‐reduction of surface oxygen species was alleviated. Therefore, the enhancement of mesoporosity by alkali treatment improved the diffusion of methane to active iron sites. The mesopore area considerably increased with alkali treatment from 28.6 to 104.2 m^2^/g (Fe‐FER‐0.3). The formation of mesopores enhanced in Fe‐FER‐0.1 and Fe‐FER‐0.2. It reduced the formation of iron oxide nanoparticles by increasing the accessibility of iron ions. On the contrary, mesopore formation was excessively increased because of damage to the zeolite structure, forming more nanoparticles in Fe‐FER‐0.3. It leaded to severe reduction in N_2_O conversion and lower methane conversion (2.4 %). The alkali treatment of Fe‐FER‐0.2 resulted in a considerable increase in DME (530 ppm *versus* 979 ppm). Additional oxidation was prevented because of the increase in silanol groups by alkali treatment. Therefore, methanol yield was enhanced. Moreover, the meso‐microporous hybrid structure allowed the product to desorb before further oxidation. In particular, DME was highly prone to diffusion and desorption in alkali‐treated zeolites because of its large molecular size. Consequently, Fe‐FER‐0.2 was the best with regard to the conversion of methane and N_2_O and selectivity of useful substances.

A comparison was conducted between loading Fe into FER using a liquid ion exchange method (Fe‐FER‐IE) and solid‐state ion exchange (Fe‐FER‐SSIE).^[49]^ The relationship between the stability of surface oxygen species and catalytic activity as a function of Fe site characterization was investigated. The micropore area was similar for both catalysts at approximately 253 m^2^/g (Table 2, entries 5–6). The micropore volume was also comparable at approximately 0.13 cm^3^/g (Table 2, entries 5–6). However, a difference in the distribution and type of Fe species was observed. Fe‐FER‐SSIE had more proton exchange with Fe cations. It also formed isolated and oligomeric extra‐framework Fe species. On the contrary, Fe‐FER‐IE primarily formed isolated framework Fe sites. The methane conversion of Fe‐FER‐SSIE and Fe‐FER‐IE improved with the increase of temperature (similar to 0.8–4.5 % in both). Fe‐FER‐IE enhanced N_2_O conversion from 7.5 % to 43.7 %, and Fe‐FER‐SSIE increased from 5.8 % to 36.6 % with the increase of temperature. Fe‐FER‐IE consumed more N_2_O when the methane conversion was similar. Fe‐FER‐IE was relatively more active in the decomposition of active oxygen species, making it easier to generate oxygen molecules. Considering that oxygen molecules do not activate methane, more N_2_O was required to achieve the same methane conversion. The release of oxygen molecules led to the deeper oxidation of intermediates, resulting in slightly less methanol and DME in Fe‐FER‐IE. Fe‐FER‐SSIE contained more binuclear extra‐framework Fe species, which made it more stable to reactive oxygen species. Fe‐FER‐SSIE was highly reactive with methane. Accordingly, more DME was generated than Fe‐FER‐IE (about 17–65 %).

Impregnation is a widely used method for preparing catalysts with alpha sites, but it has the limitation of poor dispersion of iron species. Fan et al. used freeze drying to enhance the formation of alpha sites during impregnation for synthesising Fe‐ZSM‐5. The freeze‐dried Fe‐ZSM catalyst (FeZSM‐F) showed better performance than the commonly used evaporatively dried catalyst (FeZSM‐E).^[51]^ The difference in iron distribution during drying resulted in different iron structures and catalytic activities. First, the selectivity of total oxy organics was higher for FeZSM‐F (23.6 % for FeZSM‐F, 14.2 % for FeZSM‐E). In addition, the selectivity of all single oxygenates was higher for FeZSM‐F. FeZSM‐F had a 10.9 % selectivity for methanol, 5.5 % selectivity for DME and 7.2 % selectivity for formaldehyde. The rate of oxy‐organic formation was also higher for FeZSM‐F (0.22 mmol g_cat_^−1^ h^−1^) than for FeZSM‐E (0.14 mmol g_cat_^−1^ h^−1^). FeZSM‐F had more alpha site and less large iron oxide because of the superior iron dispersion, which favoured the conversion of CH_4_ to oxygenates rather than deep oxidation. It also had less Brønsted acid contributing to deep oxidation, resulting in higher selectivity. Methane conversion was similar for both catalysts, approximately 1.07 mmol g_cat_^−1^ h^−1^. The N_2_O conversion was also not much different at 3.17 mmol g_cat_^−1^ h^−1^ for FeZSM‐F and 3.3 mmol g_cat_^−1^ h^−1^ for FeZSM‐E.

**Figure 3:**
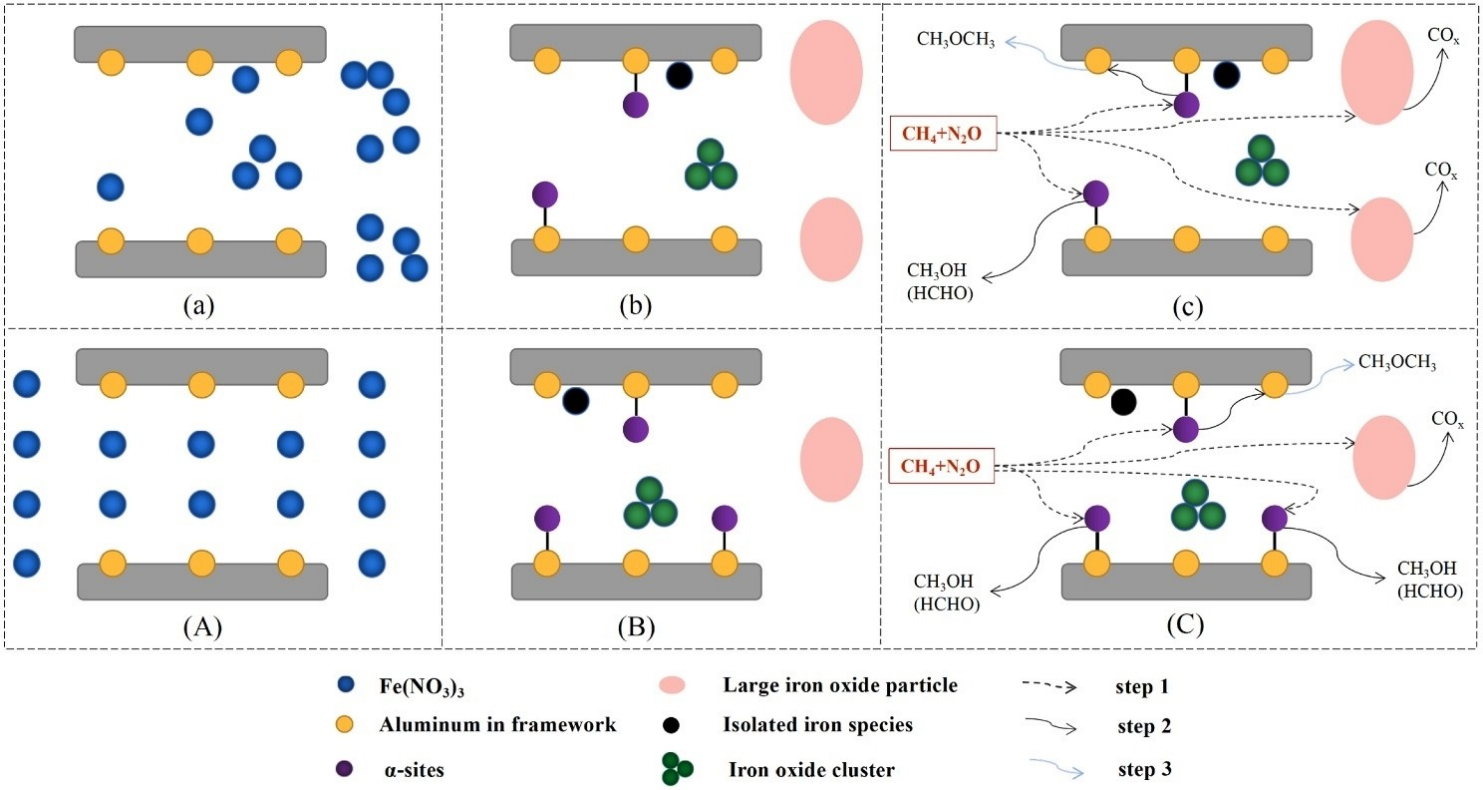
Schematic showing typical formations of iron species and reaction routes determined by different drying methods: (a) Impregnated sample after evaporation drying, (b) FeZSM‐E, (c) Reaction pathway of FeZSM‐E, (d) Impregnated sample after freeze drying, (e) FeZSM‐E, and (f) RIeaction pathway of FeZSM‐F. Reprinted from Fan et al.,^[51]^ Copyright (2019), with permission from Elsevier.

Figure 3 shows the formation of iron species and the reaction mechanism in accordance with the drying method. Figure 3a shows the migration of a large amount of Fe(NO_3_)_3_ in the pores to the surface of ZSM‐5 by capillary force through evaporative drying. Figure 3b shows the formation of large alpha‐Fe_2_O_3_ particles after calcination. Large alpha‐Fe_2_O_3_ was formed outside the pores because a considerable amount of Fe(NO_3_)_3_ was packed outside the pores (shown in Figure 3a). The remaining Fe(NO_3_)_3_ generated various forms of iron, including isolated iron species, iron oxide clusters and Fe^2+^–O‐Al. FeZSM‐F showed an evenly dispersed state of iron after drying because the sample was frozen (Figure 3d). Figure 3e represents the state after calcination. The chemical interaction between Fe(NO_3_)_3_ and framework aluminum was greatly improved, resulting in the formation of many alpha sites and fewer Brønsted acid sites. In addition, a small amount of large alpha‐Fe_2_O_3_ particles was formed because a small amount of Fe(NO_3_)_3_ remained outside the pores. Large iron oxide caused deeper oxidation to carbon oxide such as CO_2_. The generation of CO_2_ was activated due to marked amounts of iron oxide (Figure 3c). On the other hand, it could be seen that less deep oxidation occurs in FeZSM‐F (Figure 3e). In other words, it was easier to convert to oxygenates in FeZSM‐F.

Fe catalysts also have been used with other support except for zeolite. The catalytic performance of the FePO_4_ catalyst‐mediated conversion of methane to methanol using N_2_O, O_2_ and H_2_O as oxidants was compared.^[52]^ The FePO_4_ catalyst had a tridymite phase (FePO_4_‐tdm). The oxidation reaction temperature was 200–500 °C. In all cases, the methane conversion was proportional to the increase in temperature. The methane conversion was about 17 % with O_2_, 9 % with N_2_O and 1 % with H_2_O at 500 °C and a gas hourly space velocity (GHSV) of 3600 h^−1^. However, methanol selectivity was highest with N_2_O. Under the abovementioned conditions, the methanol selectivity was about 43 % with N_2_O, 30 % with H_2_O and 14 % with O_2_. Methanol selectivity was highest at low conversion rates with high flow rates. With increasing temperature, the methanol selectivity decreased because it was converted to CO_2_. The selectivity was maximum at 400 °C for all conditions. For iso‐conversion of 5 % at 400 °C, CO_2_ was the major product when using O_2_ and H_2_O. This indicated that methane underwent. Direct combustion or methoxy radical was oxidized to CO_2_ via further oxidation. The FePO_4_‐tdm‐phase catalyst showed a high activity for methanol formation with N_2_O (12.3×10^−3^ μmol_MeOH_ g_cat_^−1^ h^−1^ at 300 °C), which was higher than that with O_2_ (5.3×10^−3^ μmol g_cat_^−1^ h^−1^ at 300 °C).

The number of studies using iron‐based catalysts in combination with copper for the selective oxidation of methane is small because the properties of the Cu species favor the total oxidation of methane. However, the conversion might be improved by alteration of the catalyst properties towards co‐operation of phases. The application of bimetallic catalysts prepared via co‐loading other metals with the Fe species has been studied. Copper and iron‐based catalysts with Al_2_O_3_ as support were prepared via co‐precipitation (denoted as Cu‐Fe/Al_2_O_3_ (COP)) or hydrothermal methods (denoted as Cu‐Fe/Al_2_O_3_ (hydrothermal)).^[23]^

Cu‐Fe/Al_2_O_3_ (hydrothermal) had a wider pore‐size distribution than Cu‐Fe/Al_2_O_3_ (COP). It also had a larger pore volume (0.27 cm^3^/g *versus* 0.19 cm^3^/g) (Table 2, entries 10–11). Copper nanoparticles exhibited a hydrogen spillover effect. The activated hydrogen spilled over to Fe_2_O_3_, acting as hydrogen activation sites. In brief, the synergistic effect of copper and Fe relieved the reduction temperature of iron in bimetallic catalysts. The reduction was primarily affected by temperature, so no difference in the reduction pathway for both bimetallic catalysts was observed. The alpha sites of iron and copper carried out oxidation via the reversible redox transitions Fe^2+^↔Fe^3+^ and Cu^+1^↔Cu^2+^. On the bimetallic surface, Cu‐Fe species included isolated, oligonuclear and large agglomerates of metal oxide. Cu‐Fe/Al_2_O_3_ (hydrothermal) exhibited better catalytic performance (400 °C, GHSV 3600–7200 h^−1^, CH_4_:N_2_O=1 : 1). It exhibited a maximum methanol selectivity of about 70 % when CH_4_ conversion was 2.5 % and then decreased because of the subsequent oxidation of methane. Cu‐Fe/Al_2_O_3_ (COP) exhibited a methanol selectivity of about 50 % when CH_4_ conversion was 1.5 %. No considerable difference in methane conversion was observed between the two catalysts because of the similar surface area of the catalysts. The difference between the two catalysts for the selectivity of methanol was related to the higher metal dispersion on Cu‐Fe/Al_2_O_3_ (hydrothermal) than Cu‐Fe/Al_2_O_3_ (COP). Comparing the bimetallic and monometallic catalysts, the bimetallic catalyst had a maximum methanol selectivity at 300 °C because of its more readily reducible nature, whereas the monometallic catalyst had a maximum methanol selectivity at 400 °C. The bimetallic catalysts exhibited almost twice the selectivity compared with the monometallic catalysts. The maximum methanol selectivity of the catalysts at 4000 h^−1^ was approximately 80 % for Cu‐Fe/Al₂O₃ (hydrothermal) and 70 % for Cu‐Fe/Al₂O₃ (COP), which were about 30 % and 18 % higher than those of Cu/Al₂O₃ and Fe/Al₂O₃, respectively. In addition, the methanol yield of Cu‐Fe/Al_2_O_3_ (hydrothermal) was stable up to 40 h. When water was added to the gas feed, the water gas shift reaction was activated, thereby decreasing the CO selectivity, and increasing the CO_2_ selectivity. Water did not affect the nature of the active sites, but as the water adsorbed on the sites, the accessibility to the active sites was reduced, resulting in a decrease in selectivity. However, the reduction in selectivity was counterbalanced by an increase in conversion in the presence of water, and the yield regained its initial value. High turnover frequency (TOF) in the range of 15–18×10^−3^ s^−1^ were achieved.

The Al atoms within each zeolite framework can be considered in terms of their location in the network of channels and cages. The position of the Al tetrahedron determines the accessibility of the active sites and metal counter cations. In addition, the position of Al controls the formation of reaction intermediates in the confined void volume of the charge‐balancing cations.^[53]^ The framework Al arrangement affects the structure and speciation of exchanged metal ions and complexes, which are precursors to active sites for methane partial oxidation.^[54]^ Many strategies have been attempted to control the position of Al atoms in zeolites, and to date control methods using Na cations have been attempted in the synthesis of zeolites. On the other hand, Al distribution during the synthesis of AEI zeolite is very difficult due to harsh synthesis conditions and narrow Si/Al ratio range and has rarely been attempted. The Xiao group made the first attempt to control the AI distribution by utilising Na cations in AEI‐type aluminosilicate zeolites.^[55]^ Among *x*Cu/AEI(Na) and *x*Cu/AEI(Na free) prepared with various Cu contents, 5Cu/AEI(Na) and 5Cu/AEI(Na free) prepared with 5 mmol/L of Cu(NO_3_)_2_ exhibited excellent methanol formation rates at 350 °C. The amount of Al atoms in the catalyst, i. e., acidic properties and Cu speciation, were related to the catalytic performance. At a low Cu content (1Cu/AEI), the acidic sites was important parameter. Also, product distribution was similar regardless of the presence of Na. 5Cu/AEI(Na) had an evenly matched amount of acid and Cu species. It has similar product distribution with 1Cu/AEI(Na), but the reactants (CH_4,_ N_2_O) conversion and methanol formation rates were better. The methanol production rates and selectivity of 5Cu/AEI(Na free) continuously increased from 17.2 mmol g^−1^ min^−1^ and 21 % to 26.5 mmol g^−1^ min^−1^ and 37 %, respectively, over 300 min. 5Cu/AEI(Na) exhibited an initial methanol formation rate of 25.3 mmol g^−1^ min^−1^ and selectivity of 46 %, which decreased after 180 min with a large conversion of methanol to olefins. These reaction results were attributed to the acidic and copper features between the two catalysts. 5Cu/AEI(Na) had more dicopper species than 5Cu/AEI(Na free) and thus exhibited improved activity in the methane to methanol conversion. Moreover, 5Cu/AEI(Na) had a higher amount of acid than 5Cu/AEI(Na free). It led a faster conversion of methanol to olefins (Table 2, entries 12–13). Extra calcination at 750 °C for 10 h was performed to improve their stability (5Cu/AEI(Na)‐750, 5Cu/AEI(Na free‐750)). 5Cu/AEI(Na)‐750 reached a methanol formation rate of 27.7 μmol g^−1^ min^−1^ with 48 % selectivity and remained stable for 1500 min (350 °C, weight hourly space velocity (WHSV)=15000 mL g^−1^ h^−1^, CH_4_:N_2_O:H_2_O:Ar=10 : 10 : 2 : 3 mL/min). In addition, 5Cu/AEI(Na free)‐750 was stable, but the methanol formation rate was only ≈5 μmol g^−1^ min^−1^, and the selectivity was <7 % at the same conditions. It showed advantages over other literature results in terms of methanol formation rate and selectivity.

Memioglu group investigated the effect of mesoporosity and methanol production ability of zeolites of various frameworks, including MAZ and AEI.^[56]^ As a result, it was confirmed that Micro‐Cu‐SSZ‐39 (Cu(II)‐exchanged zeolites) had the most active methanol production ability (90 μmol g^−1^ h^−1^). The micro‐Cu‐SSZ‐39 (6.3 Si/Al, 0.22 Cu/Al) contributed to a notable continuous methanol formation rate of 499 μmol_MeOH_ g^−1^ h^−1^ and 34 % selectivity of methanol. Optimization of the N_2_O, H_2_O, N_2_O, and CH_4_ partial pressures for this catalyst was performed at 270–325 °C. For a range between 5.1 and 0.0203 MPa, the effect of N_2_O partial pressure was studied. As the partial pressure of methanol increases, selectivity decreased because of the acceleration of overoxidation. The Partial pressure of H_2_O effect was studied with the optimised N_2_O (0.0152 kPa) and CH_4_ partial pressures (0.0405 MPa) at 325 °C. H_2_O could play an auxiliary role in forming an active site including isolated Cu(II) migration. The lower partial pressures (0.0026–0.004 MPa) of H_2_O led improved methanol formation. However, higher Cu loading (Cu/Al=0.33) led to much smaller rates (76 μmol_MeOH_ g^−1^ h^−1^). Also, it could be found a rate‐determining step including methanol desorption, or generation of active site with water. Desorption of methanol was observed for the [Cu‐O‐Cu]^2+^ moiety under moisture environment. By suppressing the reaction between C_1_ intermediate and the oxidant, Overoxidation reaction rates were reduced by facilitating methanol desorption from the surface. Methanol formation decreased due to over‐oxidation at the higher H_2_O partial pressure around 0.01 MPa. It could be related with deactivation by water poisoning. Water molecules can be potential reactant competitors. The Sushkevich group studied the selective oxidation of methane to methanol using oxygen‐activated copper‐exchanged mordenite as a catalyst. They noted that when the catalyst is partially poisoned by water, at least two copper atoms in the active site are covered for each water molecule. They also noted that water molecules have a competitive adsorption relationship with methane.^[57]^

Dai′s group demonstrated the effectiveness of the active site motif structure in the oxidation of methane to methanol via Cu‐ZSM‐5. Insitu FTIR spectroscopy demonstrated that the binuclear [Cu‐O‐Cu]^2+^ site can be easily created over the neighboring Cu^+^ cationic site ([Cu]^+^‐[Cu]^+^) via interaction with N_2_O. The results show that both the monomeric [Cu]^+^ site and the binuclear [Cu‐O‐Cu]^2+^ site follow a radical mechanism that activates methane to methyl and hydroxyl radicals, contributing to the production of CH_3_OH. However, the binuclear [Cu‐O‐Cu]^2+^ site shows higher activity than the monomeric [Cu]^+^ site, with the positive effect of lowering the N_2_O decomposition dissociation barrier.^[58]^

**Figure 4:**
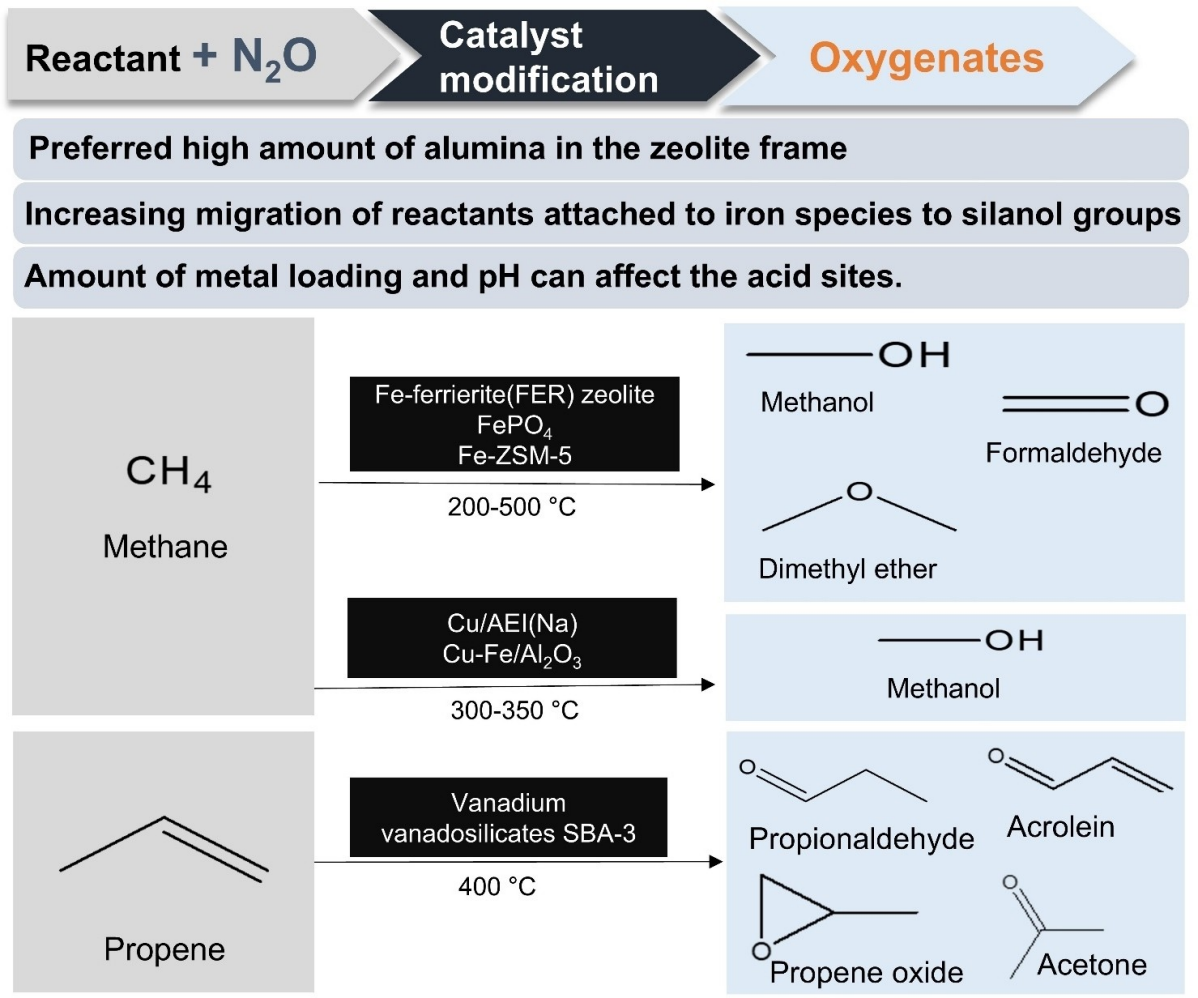
Schematic diagram of N₂O‐assisted oxygenates production.

Mesoporous silica materials with loading vanadium are preferred in N_2_O‐assisted propene epoxidation.^[59]^ Separated vanadium species have influence on superior selectivity of propene oxide (PO).^[60]^ However, existing research produced vanadium catalysts through impregnation. Janiszewska group attempted to improve catalytic activity by making well‐dispersed isolated vanadium through direct synthesis of vanadium containing SBA‐3 materials. Vanadium‐containing silica SBA‐3 mesoporous catalysts was synthesised using a hydrothermal method with NH_4_VO_3_ or VOSO_4_ as vanadium precursors under acid conditions (pH <1, 2.2, 3.1).^[61]^ The effect of various variables (pH of synthesis mixture, vanadium precursor) on product characteristics was investigated. The catalysts names were denoted as VSy‐SBA‐3 (y is pH, VOSO_4_ as precursor) or VMy‐SBA‐3 (y is pH, NH_4_VO_3_ as precursor). In the case of a sample synthesised using VOSO_4_, the vanadium content was low and similar regardless of the pH of the synthetic mixtures. Propene conversion and selectivity decrease with the increase of pH. Considering that the vanadium content was similar, the decrease in propene conversion might be due to the surface area. On the contrary, samples obtained with NH_4_VO_3_ include low vanadium. However, vanadium content increased more than five times as pH increases due to better interaction of V‐OH and Si‐OH at higher pH. by this effect, the propene conversion rate at 400 °C reached about 15 %. This was a dramatic increase compared with the case of a sample prepared with NH_4_VO_3_ at pH 1 or less (about 2.5 % conversion). NH_4_VO_3_ seemed to be a better than VOSO_4_ in molding a vanadosilicate SBA‐3 structure. Regardless of the precursor, if the pH of the synthetic mixture increased, then the surface area and pore volume decreased because silicate conditions occur faster at higher pH. This effect was more evident when using NH_4_VO_3_. Moreover, in the case of samples using NH_4_VO_3_ at high pH, the porosity accessible by external vanadium specifications was reduced, thereby reducing the pore volume and surface area. (Table 2, entries 14–16) Regardless of vanadium amount, all samples showed well distribution, isolated tetrahedrally coordinated VO_*x*_ specie. Samples with high vanadium concentration had various forms of vanadium species. More acidic sites of samples were synthesised at high pH using NH_4_VO_3_. Vanadium content was proportional to the strength of the acid sites (Lewis and Brønsted). Lewis acidity was more dominant. A higher vanadium content led to improved formation rates of PO per unit mass of catalyst per unit time (STY). However, in vanadium‐rich samples (>5 wt%), penta‐ and octahedral coordination vanadium became inactive, which had bad effect on the generation of mild electrophilic oxygen species. Therefore, TOF was small with high vanadium samples. The deviation of the propene oxide (PO) selectivity by sample was not large. VM_2_‐SBA‐3 showed a figure of about 16 % PO selectivity. In addition to PO, propionaldehyde (PA), acetone and acrolein were produced. PA was the main product at about 50 % at a low vanadium content (<1 wt%) regardless of the synthesis condition with the vanadium precursor. As vanadium content increases, carbon oxide selectivity considerably increased, and PA selectivity decreased by about 10–20 % at 400 °C. Figure 4 presents a summarized flowchart of N₂O‐assisted oxygenates production, illustrating the reactants, target products, catalysts, and key objectives of catalyst modification in this paper. Table 2 shows the characteristics of catalysts for conversion to oxygenates. Table 3 summarizes the catalytic performance of conversion to oxygenates.

**Table 3:** Catalytic performance of conversion to oxygenates.

Entry	Name	Reaction condition					Conversion(%)			Product Selectivity(%)							Ref.
		Temperature (°C)	Pressure (MPa)	Reactants	Space velocity	Time (h)	N_2_O	Methane	Propene	Methanol	Dimethyl ether (DME)	Formaldehyde	Acetone	Acrolein	Propene oxide	Propionaldehyde	
1	Fe‐ZSM‐5	350	‐	CH_4_: N_2_O=28 : 7	‐	6	7.1	1.4	‐	3.1	trace	4.7	‐		‐	‐	[46]
2	Fe‐BETA	350	‐	CH_4_: N_2_O=28 : 7	‐	6	9.1	1.3	‐	4.7	trace	6.5	‐	‐	‐	‐	
3	Fe‐FER	350	‐	CH_4_: N_2_O=28 : 7	‐	6	20	2.4	‐	21	20.6	4,8	‐	‐	‐	‐	
4	Fe‐FER‐0.2	350	‐	CH_4_: N_2_O=28 : 7	‐	6	22	2.8	‐	650 ppm	979 ppm	120 ppm	‐	‐	‐	‐	[50]
5	Fe‐FER‐IE	300–360	‐	CH_4_: N_2_O=28 : 7	‐	‐	7.5–43.7	0.8–4.5	‐	5–15	15–55	0–2.5	‐	‐	‐	‐	[49]
6	Fe‐FER‐SSIE	300–360	‐	CH_4_: N_2_O=28 : 7	‐	‐	5.8–36.6	0.8–4.4	‐	6–16	17–65	0–2.5	‐	‐	‐	‐	
7	FeZSM‐F	300	0.1	CH_4_: N_2_O=1 : 1	‐	‐	3.17 mmol⋅g^−1^⋅h^−1^	1.06 mmol⋅g^−1^⋅h^−1^	‐	10.9 mol%	5.5	7.2	‐	‐	‐	‐	[51]
8	FeZSM‐E	300	0.1	CH_4_: N_2_O=1 : 1	‐	‐	3.3 mmol⋅g^−1^⋅h^−1^	1.07 mmol⋅g^−1^⋅h^−1^	‐	9.1	1.9	3.2	‐	‐	‐	‐	
9	FePO_4‐_tdm	200–500	0.1	CH_4_: N_2_O=1 : 1	3600 h^−1^	5	‐	0.5–9	‐	ab. 5–42.5		~25 (yield)	‐	‐	‐	‐	[52]
10	Cu‐Fe/Al_2_O_3(_COP)	300	0.1	CH_4_: N_2_O=1 : 1	4000 h^−1^	~40	‐	1.6	‐	ab.70	‐	‐	‐	‐	‐	‐	[23]
11		300	0.1	CH_4_:N_2_O:H_2_O=1 : 1:0.3	4000 h^−1^	~40	‐	2.5	‐	ab.50	‐	‐	‐	‐	‐	‐	
12	Cu‐Fe/Al_2_O_3_ (hydrothermal)	300	0.1	CH_4_: N_2_O=1 : 1	4000 h^−1^	~40	‐	1.8	‐	ab.80	‐	‐	‐	‐	‐	‐	
13		300	0.1	CH_4_:N_2_O:H_2_O=1 : 1:0.3	4000 h^−1^	~40	‐	2.5	‐	ab.65	‐	‐	‐	‐	‐	‐	
14	5Cu/AEI(Na) −750	350 °C	‐	CH_4_:N_2_O:H_2_O=10 : 10 : 2	15000 mL/g	‐	~0.5	~1	‐	48	‐	‐	‐	‐	‐	‐	[55]
15	5Cu/AEI(Na free) −750	350 °C	‐	CH_4_:N_2_O:H_2_O=10 : 10 : 2	15000 mL/g	‐	~8.5	~2	‐	~7	‐	‐	‐	‐	‐	‐	
16	Micro‐Cu‐SSZ‐39	325	0.1	0.0405 MPa CH_4_, 0.0152 MPa N_2_O, 0.004 MPa H_2_O	10400 h^−1^	‐	0.74	0.28	‐	34	57 μmol⋅g^−1^⋅h^−1^	‐	‐	‐	‐	‐	[56]
19	VS1‐SBA‐3	400	0.1	Propene:N_2_O=1 : 15	3420 mL/ h/g		‐	‐	5	‐	‐	‐	10	16	15	45	[61]
20	VM1‐SBA‐3	400	0.1	Propene:N_2_O=1 : 15	3420 mL/h/g		‐	‐	2.5	‐	‐	‐	18	11	12	50	
21	VM2‐SBA‐3	400	0.1	Propene:N_2_O=1 : 15	3420 mL/h/g		‐	‐	15	‐	‐	‐	8	13	16	17	

## 4 Hydrocarbon Products

In this section, the production of various hydrocarbons, primarily C_2_ hydrocarbon, including ethylene and ethane, using N_2_O as an oxidising agent is discussed. Ethane can be converted primarily to ethylene using N_2_O.^[62]^ Ethane is the second primary component of shale gas.^[63]^ The dehydrogenation of ethane with N_2_O is an exothermic process, which requires low energy consumption.^[64]^ Propylene is also one of the products that can be obtained through N_2_O assisted oxidation from propane. Propylene has been manufactured from fossil raw materials, light olefins in the naphtha steam pyrolysis process. Naphtha steam pyrolysis process is a high energy consumption process because it is carried out at about 800 °C and separation of olefins is done at a temperature as low as −100 °C.^[65]^ In other words, this is a process that requires a more energy‐efficient and eco‐friendly alternative.

Oxidative dehydrogenation of ethane (ODH) is considered as one of the attractive processes mediated ODH and there is a lack of studies investigating the structural effects of metal entities in the dehydrogenation of ethane. Xu et al. identified the effect of reduction temperature on Ni‐Fe species supported on mesoporous alumina.^[66]^ In addition, they elucidated the impact of these modifications on the performance of oxidative dehydrogenation (ODH). Before reduction, Ni‐Fe‐Al_2_O_3_ formed H_1_ hysteresis loop and type IV isotherm. It had considerably small crystalline species and no isolated nickel oxide or iron oxide particles, exhibiting a homogeneous distribution of Ni/Fe oxide species. In Ni‐Fe‐Al_2_O_3_‐H400 (reduction at 400 °C), Fe existed in the form of FeO_x_ and Ni existed in the form of Ni^2+^‐based oxide. The Ni and Fe‐based sites were in close proximity, which could lead to active electron exchange between the two sites. With more intensive reduction at 600 °C (Ni‐Fe‐Al_2_O_3_‐H600), the subsequent reduction and movement of Ni^2+^ to the surface resulted in the generation of isolated Ni nanoparticles. In addition, more Fe^3+^‐based oxides were converted to FeOX species, and they exhibited high FeO_*x*_ dispersion. Ni‐Fe‐Al_2_O_3_‐H400 and Ni‐Fe‐Al_2_O_3_‐H600 had N_2_O conversion up to 90 % at 400 °C. This N_2_O conversion was nine times higher than that of catalysts before reduction, indicating that the FeO_x_ species in Ni‐Fe‐Al_2_O_3_‐H400 Ni‐Fe‐Al_2_O_3_‐H400 and Ni‐Fe‐Al_2_O_3_‐H600 considerably contributed to the enhancement of N_2_O conversion. Given the structural differences of the catalysts, ethane conversion and ethylene selectivity were higher in Ni‐Fe‐Al_2_O_3_‐H400 compared with Ni‐Fe‐Al_2_O_3_‐H600 Ni‐Fe‐Al_2_O_3_‐H400. The conversion of C_2_H_6_ and the selectivity of C_2_H_4_ was about 32 % and 59 %, respectively, in Ni‐Fe‐Al_2_O_3_‐H400 at 400 °C. On the one hand, the C_2_H_6_ conversion and C_2_H_4_ selectivity in Ni‐Fe‐Al_2_O_3_‐H600 were 18 % and 40 %, respectively, which were lower than those in Ni‐Fe‐Al_2_O_3_‐H400. This result was likely due to the reduced Ni sites available for ethane dehydrogenation in Ni‐Fe‐Al_2_O_3_‐H600.

**Figure 5:**
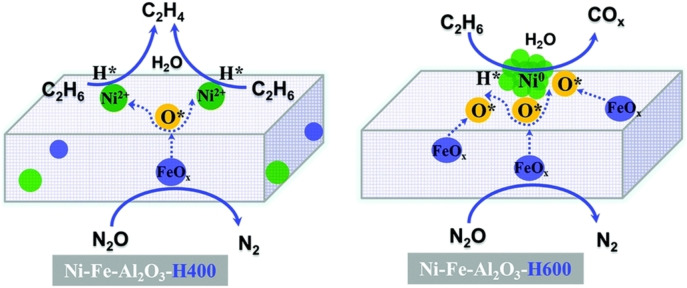
Suggested reaction schemes for the ethane oxidative dehydrogenation reaction over the Ni‐Fe‐Al_2_O_3_‐H400 and Ni‐Fe‐Al_2_O_3_‐H600 catalysts. Reproduced from Xu et al.^[66]^ with permission from the Royal Society of Chemistry.

Mesoporous alumina has uniform pores, high surface area, and good pore size distribution.^[67]^ Mesoporous alumina is a catalyst support that can improve the dispersion of active metal species.^[68]^ Cancino‐Trejo group demonstrated that NiAl_2_O_4_, NiFe_2_O_4_, and FeAl_2_O_4_ on Ni‐Fe alumina catalysts were active sites in the ODH of ethane during carbon dioxide‐mediated dehydrogenation of ethane.^[69]^ However, few have been applied to N_2_O. Ni‐Fe‐Al_2_O_3_‐H600 produced several oxygen species because of the high amount of FeO_*x*_ sites, which was effective in the decomposition of N_2_O. On the contrary, the Ni sites decreased ethane dehydrogenation because of the further reduction of Ni^2+^ and the generation of nano scale Ni species. Figure 5 shows the ODH reaction diagram of Ni‐Fe‐Al_2_O_3_‐H400 and Ni‐Fe‐Al_2_O_3_‐H600. In Ni‐Fe‐Al_2_O_3_‐H600, the number of oxygen species was greater than the number of Ni sites. On the contrary, in Ni‐Fe‐Al_2_O_3‐_H400, the ethane dehydrogenation was properly accelerated because of the high dispersion of Ni^2+^ oxide. In Ni‐Fe‐Al_2_O_3_‐H400, Fe sites that activated N_2_O decomposition were located near the Ni sites. Hydrogen from dehydrogenation was properly consumed by oxygen species generated via N_2_O decomposition, which led to an improved ethylene yield. Consequently, Ni‐Fe‐Al_2_O_3_‐H400 had a better catalytic performance. At 400 °C, an ethylene yield of 18 % was achieved, which was about three times that of Ni‐Fe‐Al_2_O_3‐_H600.^[66]^

Ni‐Al layer double hydroxides (LDH) was applied to the oxidation dehydrogenation reaction of ethane. LDH has recently attracted attention due to its excellent dispersion, large surface area, good thermal stability, and flammability resistance.^[70]^ Conventional NiO catalysts have a large amount of abundant electrophilic oxygen (O^−^) species on their surface,^[71]^ enabling C‐H activation.^[72]^ However, at the same time, it causes deep oxidation.^[73]^ Therefore, co‐loading with other metals is one research direction to improve ethylene selectivity. For the first time, Al mixed oxides (Ni_x_Al‐MO) derived from LDH was applied to the ODH of ethane.^[74]^ Ni_3_Al‐MO had a smaller NiO particle size (4 nm for Ni_3_Al‐MO, 34 nm for NiO/Al_2_O_3_) than NiO/Al_2_O_3_, whereas the Ni–Al mixed oxide prepared from LDH had a better binding of Al cations to the NiO lattice. The adsorption of reactive oxygen species occurred in Ni_3_Al‐MO due to the large specific surface area (170 m^2^/g). These oxygen species were easily generated through the decomposition of N_2_O during the ODH reaction (Table 4, entry 3).

**Table 4:** Characteristics of catalysts for conversion to hydrocarbons.

	Catalyst	Metal loading			Heat treatment	Sieve (mesh)	Concentration of base sites (μmol/g)				Surface area (m^2^/g)	Ref.
		Method	Metal	Ratio (wt %)			Weak	Intermediate	Strong	Total		
1	Ni‐Fe‐Al_2_O_3_‐400	Evaporation‐induced self‐assembly (EISA) technique	Nickel nitrate hexahydrate, Ferric nitrate nonahydrate,	Ni (5.6), Fe (1.6)	400–600 °C; 480 min; Further reduction in 5 vol % H_2_ at 400 °C for 240 min	20–40	‐	‐	‐	‐	‐	[66]
2	Ni‐Fe‐Al_2_O_3_‐600	EISA technique	Nickel nitrate hexahydrate, Ferric nitrate nonahydrate,	Ni (5.6), Fe (1.6)	400–600 °C; 480 min; Further reduction in 5 vol % H_2_ at 600 °C for 240 min	20–40	‐	‐	‐	‐	‐	
3	Ni_3_Al‐MO	Co‐precipitation	Nickel (II) nitrate hexahydrate, Aluminium nitrate nonahydrate	NiO (80)	500 °C	‐	‐	‐	‐	‐	170	[74]
4	NiO/Al_2_O_3_	Wetness impregnation	Nickel (II) nitrate	NiO (80)	500 °C	‐	‐	‐	‐	‐	‐	
5	Mn‐Na_2_WO_4_/SiO_2_	Incipient wetness impregnation	Sodium tungstate, Manganese (II) nitrate, Silicon dioxide	‐	800 °C	355–450 μm	‐	‐	‐	‐	‐	[80]
6	m‐Sm_2_O_3_	Wet impregnation	‐	‐	800 °C; 480 min	40–60	8.2	18.7	‐	26.9	5.3	[81]
7	15CaO/m‐Sm_2_O_3_	Wet impregnation	Calcium nitrate tetrahydrate	Cu (15)	800 °C; 480 min	40–60	‐	267	‐	267	4.8	
8	6Li/m‐Sm_2_O_3_	Wet impregnation	Lithium nitrate	Li (6)	800 °C; 480 min	40–60	‐	68	328	399	4.9	
9	4Mn/10Na/m‐Sm_2_O_3_	Wet impregnation	Manganese (II) nitrate hydrate, Sodium tungstate dihydrate	Mn (4), Na (10)	800 °C; 480 min	40–60	101	14	54	169	5.8	
10	Ag‐Fe_2_O_3_‐cenospheres	Electroless plating of silver, iron deposited by thermal decomposition of iron pentacarbonyl Fe(CO)_5_	Iron pentacarbonyl Fe(CO)_5_, methanolic solution of silver nitrate	Silver (7.10 wt%) and hematite (12.51 wt%)	600 °C	‐	‐	‐	‐	‐	‐	[19]
11	1 %Cu‐BEA	Impregnation	Cupric Nitrate trihydrate	Cu (1)	550 °C; 360 min.	40–60					‐	[83]
12	1 %CuO‐SiO2	Impregnation	Cupric Nitrate trihydrate	Cu (1)	550 °C; 360 min.	‐						
13	NiAl‐B	Co‐precipitation method & Addition of SDS (NaC_12_H_25_SO_4_)	Nickel(II) nitrate hexahydrate,,Aluminum nitrate nonahydrate	Na (1)	600 °C; 180 min	‐					90	[75]
14	NiAl‐S1	Co‐precipitation method & Addition of SDS (NaC_12_H_25_SO_4_)	Nickel(II) nitrate hexahydrate,,Aluminum nitrate nonahydrate	Na (1.1)	600 °C; 180 min	‐					128	
15	NiAl‐S2	Co‐precipitation method & Addition of SDS (NaC_12_H_25_SO_4_)	Nickel(II) nitrate hexahydrate,Aluminum nitrate nonahydrate	Na (1)	600 °C; 180 min	‐					122	

**Figure 6:**
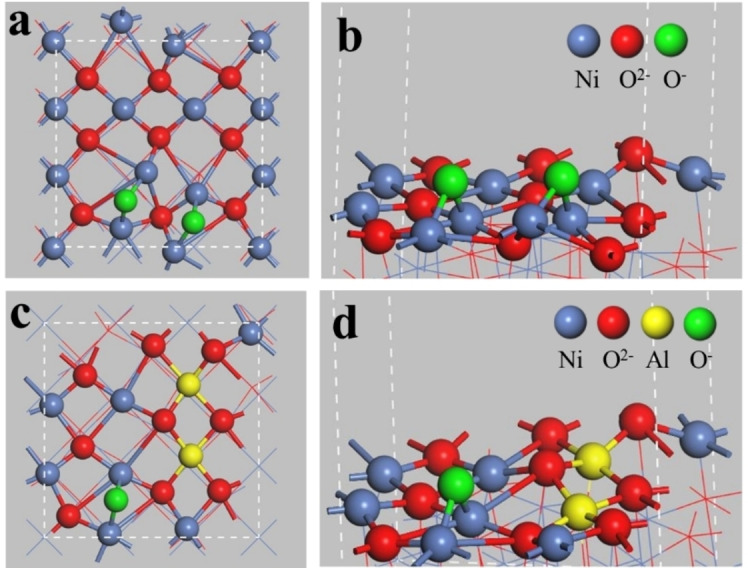
Models of pure NiO (a, b) and Al‐doped NiO (c, d) from top and side views. Reprinted from Zhou et al.,^[74]^ Copyright (2019), with permission from Elsevier.

Figure 6 shows the models of pure NiO and Al‐doped. Figure 4a and b represent NiO/Al_2_O_3_, and Figure 6 **c and d** represent Ni_3_Al‐MO derived from LDH. The formation of the Ni–O‐Al bond in Ni_3_Al‐MO resulted in the strong bonding of Ni and O species. Doped Al cations enhanced the Ni–O bond and reduced the O species on theNi_3_Al‐MO surface. Al greatly dispersed Ni and Al cations, leading to the improved isolation of O species. Isolated O species serve as active sites. Therefore, Compared with NiO/Al_2_O_3_ with the same NiO loading, Ni_3_Al‐MO showed higher ethylene selectivity (70–98 % versus 51–65 %) and higher ethane conversion (0– 25 % versus 0–35 %) when W/F (catalyst weight/feed gas flow rate) was 0.6 g s^−1^ m^−3^.

**Figure 7:**
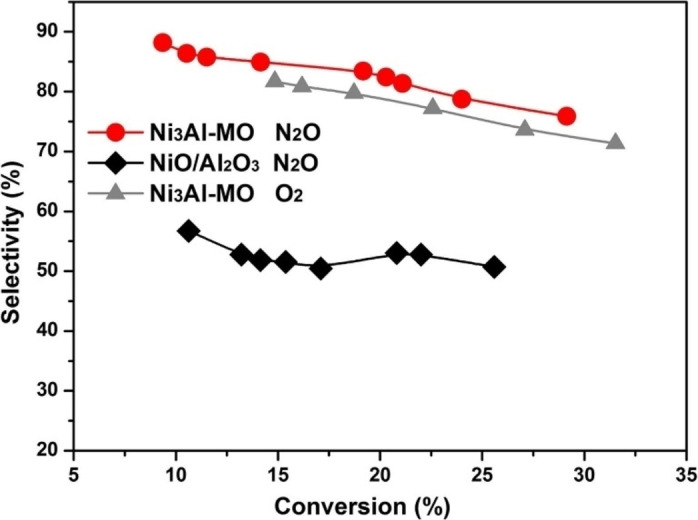
Ethylene selectivity as a function of ethane conversion at different space velocities. Reaction condition: 440 °C, C_2_H_6_:N_2_O:He=1 : 1 : 48 or C_2_H_6_:O_2_:He=2 : 1 : 97, 0.05–0.6 g s^−1^ cm^−3^. Reprinted from Zhou et al.,^[74]^ Copyright (2019), with permission from Elsevier.

Figure 7 shows a comparison of ethylene selectivity as a function of ethane conversion. Ni_3_Al‐MO had ethylene selectivity ranging from 76 % to 88 %. This value was approximately 30 % higher than that of NiO/Al_2_O_3_ at a similar conversion of ethane. When Ni_3_Al‐MO was applied to ODH with O_2_ as the oxidant, it showed 5 % less ethylene selectivity than when N_2_O was used as the oxidant. Therefore, using N_2_O as the oxidant was more beneficial than using O_2_ for ODH. Ni_3_Al‐MO has a TOF of 99 h^−1^ at 460 °C, which was better than other literature. In addition, Ni_3_Al‐MO exhibits remarkable stability. After 2880 min of operation, no deactivation was observed for either the conversion of ethane or the selectivity of ethylene.

**Figure 8:**
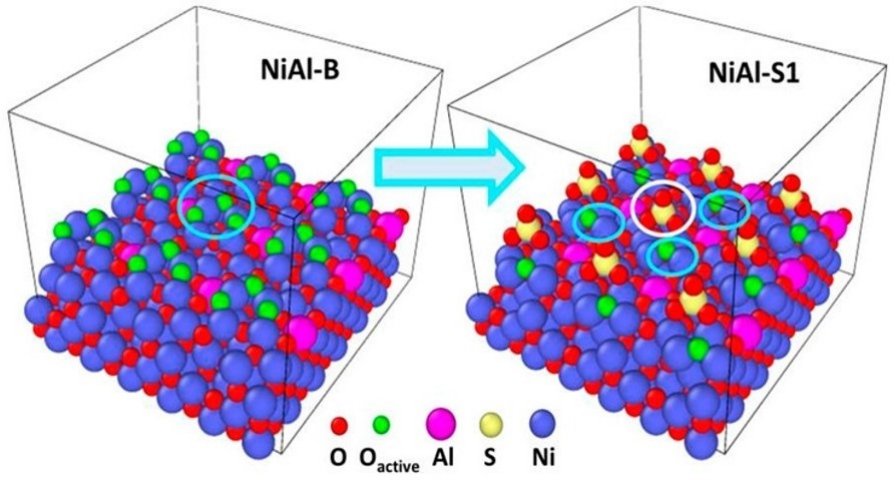
Structures of NiAl‐B and NiAl‐S1 catalysts with surface electrophilic oxygen species. Reprinted from Zhou et al.^[75]^ Copyright (2020) American Chemical Society.

Peroxidation by electrophilic surface species of NiO was due to the poor spatial distribution of oxygen species. It is therefore important to distinguish the types of oxygen species that led more reactive ODH and ethylene production. By using the anionic groups of sulphate modifiers to modulate the NiO surface oxygen species, Zhou group achieved 100 % selectivity of ethylene at 10 % ethane conversion.^[75]^ This was a significant improvement over the previously reported performance of NiO‐based catalysts. Ultrathin NiAl layered double hydroxide (LDH) was preferred as a precursor for better dispersion of metal species. The catalyst obtained with 0.05 mol of surfactant NaC_1_2H_25_SO_4_ was named NiAl‐S1, the catalyst with 0.003 mol of NaC_12_H_25_SO_4_ was named NiAl‐S2, and the catalyst without surfactant was named NiAl‐B. Electrophilic oxygen species were separated on NiAl‐S1 catalysts, while they were mainly adjacent on NiAl‐B catalysts. The addition of a sulphate modifier enhances the production of more isolated oxygen species by steric and electron extracting from wider range of Ni^3+^ species. Figure 8 shows the isolated and adjacent oxygen species on the NiAl‐B and NiAl‐S1 catalyst surfaces.

The higher Ni^3+^/Ni ^2+^ ratio led to more larger amount of separated oxygen species. Almost 100 % of isolated oxygen species derived with a Ni ^3+^ /Ni^2+^ ratio of 2.6, indicating 100 % selectivity of ethylene. In short, higher amount of isolated electrophilic oxygen species could enhance the selectivity of ethylene. Ethylene selectivity of NiAl‐B, NiAl‐S2, and NiAl‐S1 catalysts was ≈78 %, ≈91 %, and almost 100 %, respectively at 560 °C. At a higher ethane conversion of 52 %, NiAl‐S1 still showed a superior selectivity of 81 %. Due to the strong binding of ethylene intermediates, the C‐H bond of ethane was exposed to excessive cleavage with a lot of closed oxygen species, leading to peroxidation. In comparison, the isolated oxygen species were found to favour the adequate cleavage of the C‐H bond of ethane to form ethylene was accelerated by separated oxygen species. Also, desorption of ethylene from catalyst became easy. The adsorption of N_2_O on the NiAl‐S1 catalyst was dissociative, generating isolated oxygen species. It meaned good recyclability of the isolated oxygen species on the NiAl‐S1 catalyst. NiAl‐S1, NiAl‐S2 and NiAl‐B also had good stability towards the ODH of ethane by N_2_O. The application of sulphate molecules to promote the isolation of oxygen species can be well applied to other catalytic materials, which is a promising way to obtain better ethylene selectivity in the ODH of ethane.

The oxidative coupling of methane (OCM) reaction is a way to obtain ethylene and ethane from methane. The use of N_2_O in the OCM reaction has been found to have several advantages over the use of O_2_ in the process. For example, the use of N_2_O suppresses the production of gas‐phase methyl peroxo species, which are produced in the presence of O_2._ This suppression reduces CO₂ production and increases selectivity^[76]^ Numerous researchers have been working on the development of catalysts and processes that can reach sufficient ethylene and ethane yields. However, there is still a need to achieve higher performance. To achieve this, Researchers modify existing catalysts or discover new ones.

In the oxidative coupling of methane reactions, Mn‐Na_2_WO_4_/SiO_2_ catalysts are one of the promising candidates based on their high activity and selectivity as well as their long‐term stability.^[77]^ However, most of the oxidative coupling of methane reactions with this catalyst have been studied using oxygen. N_2_O, unlike O_2_, can generate monatomic oxygen species.^[78]^ Studies have shown that monoatomic oxygen species lead to higher selectivity for hydrocarbon production.^[79]^ Nevertheless, the use of N_2_O in oxidative coupling of methane reactions over Na_2_WO_4_/SiO_2_ catalysts has been much less common. Aydin et al. investigated the effects of water introduction and the type of oxidant (N_2_O or O_2_) on the differences in the OCM using Mn‐Na_2_WO_4_/SiO_2_.^[80]^ The oxidant type affected the phase composition of the catalyst because different oxidants produce different types of oxygen species. The fresh catalyst contained the tetragonal phase (α‐cristobalite) of SiO_2_. This phase did not exist after performing the N_2_O‐OCM or O_2‐_OCM reaction in the presence of water. However, the hexagonal phase (α‐quartz) of SiO_2_ appeared. The MnSiO_3_ phase could be identified after N_2_O‐OCM, whereas the Mn_7_O_8_(SiO_4_) phase was present in the spent catalyst after O_2‐_OCM (without and with cofed water). N_2_O re‐oxidised the reduced catalyst slower than O_2_, reducing the surface density (spatial separation) of lattice oxygen species in N_2_O‐OCM. N_2_O reduced surface bimetallic oxygen species that converted directly CH_4_ to CO_2_. Therefore, using N_2_O as an oxidant instead of O_2_ inhibited the direct oxidation of CH_4_ to CO_2_ and increased C_2_ hydrocarbon selectivity. At 800 °C, the selectivity of C_2_^+^ hydrocarbons were about 80 % in N_2_O‐OCM and about 60–78 % in O_2_‐OCM. Methane conversion and C_2_ hydrocarbon selectivity in N_2_O‐OCM with H_2_O was improved because H_2_O converted surface bimetallic oxygen species to monotonic species. The strength of these effects was less than that in O_2‐_OCM. In dry N_2_O‐OCM, the methane conversion reached 7.5 %, while in wet N_2_O‐DCM, it was larger at 10 %. When N_2_O and H_2_O were injected together, the complementary positive effects of H_2_O and N_2_O allow the control of bimetallic species, which considerably improves the selectivity of C_2_^+^ hydrogen. The selectivity of C_2+_ hydrocarbons was 75–85 % in dry N_2_O‐OCM and 83–90 % in wet N_2_O‐OCM.

**Figure 9:**
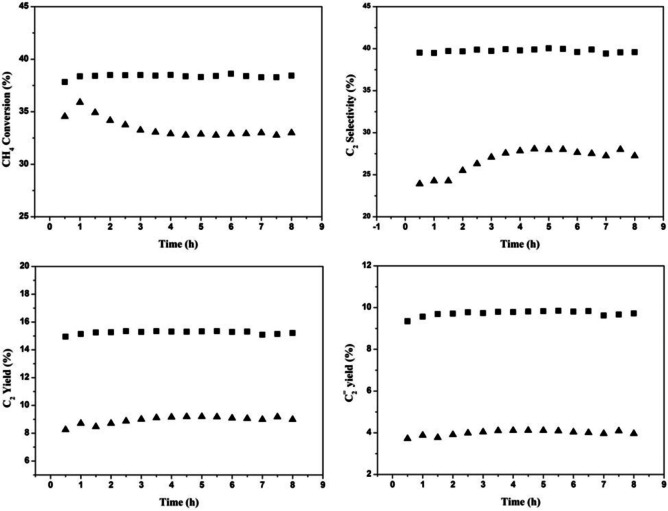
Results of short‐term stability testing of 15CaO/m‐Sm_2_O_3_ with O_2_ (▴) and N_2_O (▪). Catalyst: 400 mg (without dilution), GHSV: 7500 ml/g/h, CH_4_/O=1, *T*=780 °C, *P*=0.1 MPa. Reprinted from Özdemir et al.,^[81]^ Copyright (2018), with permission from Taylor & Francis.

Lithium, sodium, and calcium loading on cubic Sm_2_O_3_ has been performed and confirmed that each can be a candidate for an effective promoter. However, the effect of Li_2_O or CaO loading on Sm_2_O_3_ has been investigated in very few studies in the literature. Ozdemir et al. observed the effect of Li, Mn/Na_2_WO_4_ and CaO on M‐Sm_2_O_3_, which has a monoclinic phase.^[81]^ It was used for the conversion of methane to C_2_ hydrocarbon. Compared with M‐Sm_2_O_3_, basic sites increased when metals were loaded. In particular, strong basic sites considerably increased when Li was loaded (Table 4, entry 8). When CaO was loaded, intermediate basic sites were considerably increased (Table 4, entry 7). In general, except for 6Li/M‐Sm_2_O_3_, the total basicity increased with the addition of metals. This indicate that the formation of SmLiO_2_ led to a decreased Li_2_O and Li_2_CO_3_ on the 6Li/M‐Sm_2_O_3_ surface. When Mn/Na_2_WO_4_ was loaded onto M‐Sm_2_O_3_, the Sm_2_‐XMn_X_O_3_ phase was produced, thereby enhancing methane oxidation and reducing C_2_ selectivity, independent of the oxidant or loading amount. Cubic Sm_2_O_3_ was detected in all catalysts after the activity tests. When O_2_ was used for 15CaO/M‐Sm_2_O_3_, monoclinic samaria (M‐Sm_2_O_3_) was mainly converted to c‐Sm_2_O_3_. However, when N_2_O was used, the mixture of Sm(OH)_3_ and c‐Sm_2_O_3_ could be detected. Using the M‐Sm_2_O_3_ catalyst, CH_4_ and N_2_O conversion rates were comparable to those obtained using O_2_ at 740 °C (approximately 31.5 % of CH_4_ conversion and 96.8 % of N_2_O conversion), but almost twice the C_2_ selectivity was achieved using N_2_O (16.6 % of C_2_ selectivity using O_2_ and 35 % of C_2_ selectivity using N_2_O). This result indicated that M‐Sm_2_O_3_ was highly active in the decomposition of N_2_O, which was advantageous when using N_2_O as an oxidant. Among Li, Mn‐Na_2_WO_4_ and CaO, the best C_2_ yield was obtained when CaO was added. The highest C_2_ yield was 13.4 % at 780 °C using a 15 wt% CaO/M‐Sm_2_O_3_ catalyst. The C_2_ selectivity values were less than 24.7 % for the M‐Sm_2_O_3_ catalyst loaded with Mn‐Na_2_WO_4_ when N_2_O was used as an oxidant at 740 °C. Compared with M‐Sm_2_O_3_, CH_4_ conversion and N_2_O conversion decreased from 31.5 % to 12–22.5 % and from 96.8 % to 70.5–93.9 %, respectively. CH_4_ conversion and N_2_O conversion decreased from 31.5 % to 12–22.5 % and from 96.8 % to 70.5–93.9 %, respectively, when Mn‐Na_2_WO_4_ was used compared with M‐Sm_2_O_3_. At 740–820 °C, the Li‐doped M‐Sm_2_O_3_ catalyst showed the lowest reactant conversion among the tested samples (5.6–26 % for CH_4_ conversion and 42.2–73.4 % for N_2_O conversion). However, it had the highest C_2_ selectivity of the samples (49.7–70.1 %), resulting in a higher C_2_ yield (≈12.9 %) than the addition of Mn‐Na_2_WO_4_. When N_2_O was used, the C_2_ selectivity with similar CH4 conversion was high, and the selectivity of carbon oxide was low because of differences in their ability to transfer active O species. Li and CaO‐impregnated M‐Sm_2_O_3_ catalysts had similar trends. The ethylene selectivity was enhanced for c‐Sm_2_O_3_, M‐Sm_2_O_3_, Mn/Na_2_WO_4_ and CaO catalysts but lower for Li‐impregnated catalysts (Table 5, entries 7–10). For 15CaO/M‐Sm_2_O_3_, the ethylene yield reached 9.9 % (at 820 °C). Stability experiments at 780 °C indicated that the 15CaO/M‐Sm_2_O_3_ catalysts were quite stable against O_2_ and N_2_O, and the formation of Sm(OH)_3_ caused by the use of N_2_O did not negatively affect the catalyst stability (Figure 9). After the stability test using N_2_O, no material loss was found on 15CaO/M‐Sm_2_O_3_. On the contrary, 6Li/M‐Sm_2_O_3_ had low stability because of the loss of Li. Therefore, the addition of LI_2_O could improve the performance but could result in low stability.

**Table 5:** Catalytic performance of conversion to hydrocarbons.

Entry^a^	Reaction condition						Reactant conversion (%)				Product selectivity					Ref.
	Loading	Temperature (°C)	Atmosphere	Flow rate (mL/min)	GHSV	Pressure (MPa)	N_2_O	C_2_H_6_	C_3_H_8_	CH_4_	Ethylene	Ethane	C_2_ hydrocarbon	C_2+_ hydrocarbon	Propylene	
1	250 mg	400	C_2_H_6_: N_2_O=10 : 10	25			90	32			59					[66]
2	250 mg	400	C_2_H_6_: N_2_O=10 : 10	25	‐	‐	93	18		‐	40	‐	‐	‐	‐	
3	0.3 g	260–480	C_2_H_6_: N_2_O=1 : 1	30	0.6 g/s/m^3^	‐	0–100	0–25		‐	51–65	‐	‐	‐	‐	[74]
4	0.3 g	260–481	C_2_H_6_: N_2_O=1 : 1	30	0.6 g/s/m^3^	‐	0–100	0–35		‐	70–98	‐	‐	‐	‐	
5	0.3 g	600	C_2_H_6_: N_2_O=2 : 2	30	0.02–2.4 g/s/m^3^	0.1					85					[75]
6	0.3 g	600	C_2_H_6_: N_2_O=2 : 2	30	0.02–2.4 g/s/m^3^	0.1		10			100					
7	0.3 g	600	C_2_H_6_: N_2_O=2 : 2	30	0.02–2.4 g/s/m^3^	0.1					95					
8	7–700 mg	775–825	CH_4_: N_2_O=40 : 10	30	‐	0.12	‐	‐		~7.5	17–33	35–63	‐	75–85	‐	[80]
9	7–700 mg	775–825	CH_4_: N_2_O: H_2_O=40 : 10 : 30	30	‐	0.12	‐	‐		~10	20–43	30–60	‐	83–90	‐	
10	200 mg	740–820	CH_4_: N_2_O=10 : 10	‐	7500 mL/g/h	‐	96.8–99.9	‐		31.5–36.1	‐	‐	29–35	‐	‐	[81]
11	200 mg	740–820	CH_4_: N_2_O=10 : 10	‐	7500 mL/g/h	‐	92.4–99.5	‐		32.4–37.4	‐	‐	34–40.7	‐	‐	
12	200 mg	740–820	CH_4_: N_2_O=10 : 10	‐	7500 mL/g/h	‐	46.9–73.4	‐		9.9–26	‐	‐	49.7–70.1	‐	‐	
13	200 mg	740–820	CH_4_: N_2_O=10 : 10	‐	7500 mL/g/h	‐	70.5–92.5	‐		12.7–26	‐	‐	24.7–25.7	‐	‐	
14	0.1 g	550	C_3_H_8_: N_2_O=10 : 10	21	12000 h^−1^		69.4		31.5						74.5	[83]
15	0.1 g	550	C_3_H_8_: N_2_O=10 : 10	21	12000 h^−1^		27.2		3.5						43.5	

^a^ Entry number in Table 4.

N_2_O has also been reported to be an effective oxidizing agent to produce propylene through the oxidative dehydrogenation process of propane. Among zeolites such as Y, MFI, FAU, and MOR, the BEA type is one of the preferred zeolite types for decomposition of N_2_O.^[82]^ The N_2_O‐ODHP was studied over the Cu‐BEA zeolite with various active site foams such as monomeric [Cu]^+^, dimeric [Cu–Cu]^2+^, and distant [Cu]^+^–[Cu]^+^ sites.^[83]^ Controlling the Cu active site distance (Cu–Cu) was notable method for high‐efficient Cu‐zeolite type for the N_2_O‐ODHP. Dimeric [Cu–Cu]^2+^ with a Cu–Cu distance of 4.91 Å, was beneficial for the N_2_O decomposition, but it was not efficient for βH hydrogenation due to high barrier of 2.15 eV. Monomeric [Cu]^+^ was active for αH dehydrogenation, but it requires a high energy in N_2_O decomposition and βH dehydrogenation. Distant [Cu]^+^–[Cu]^+^ with the Cu–Cu distance of 5.82 Å was favorable for N_2_O decomposition, αH and βH dehydrogenation. 1 % Cu‐BEA showed better C_3_H_8 _conversion (31.5 %) and C_3_H_6_ selectivity (74.5 %) than the other catalysts (1 % Fe‐BEA, 1 % Fe‐ZSM‐5, and1 % CuO‐SiO_2_). It means CuO was not high‐impact active sites for the N_2_O‐ODHP.

**Figure 10:**
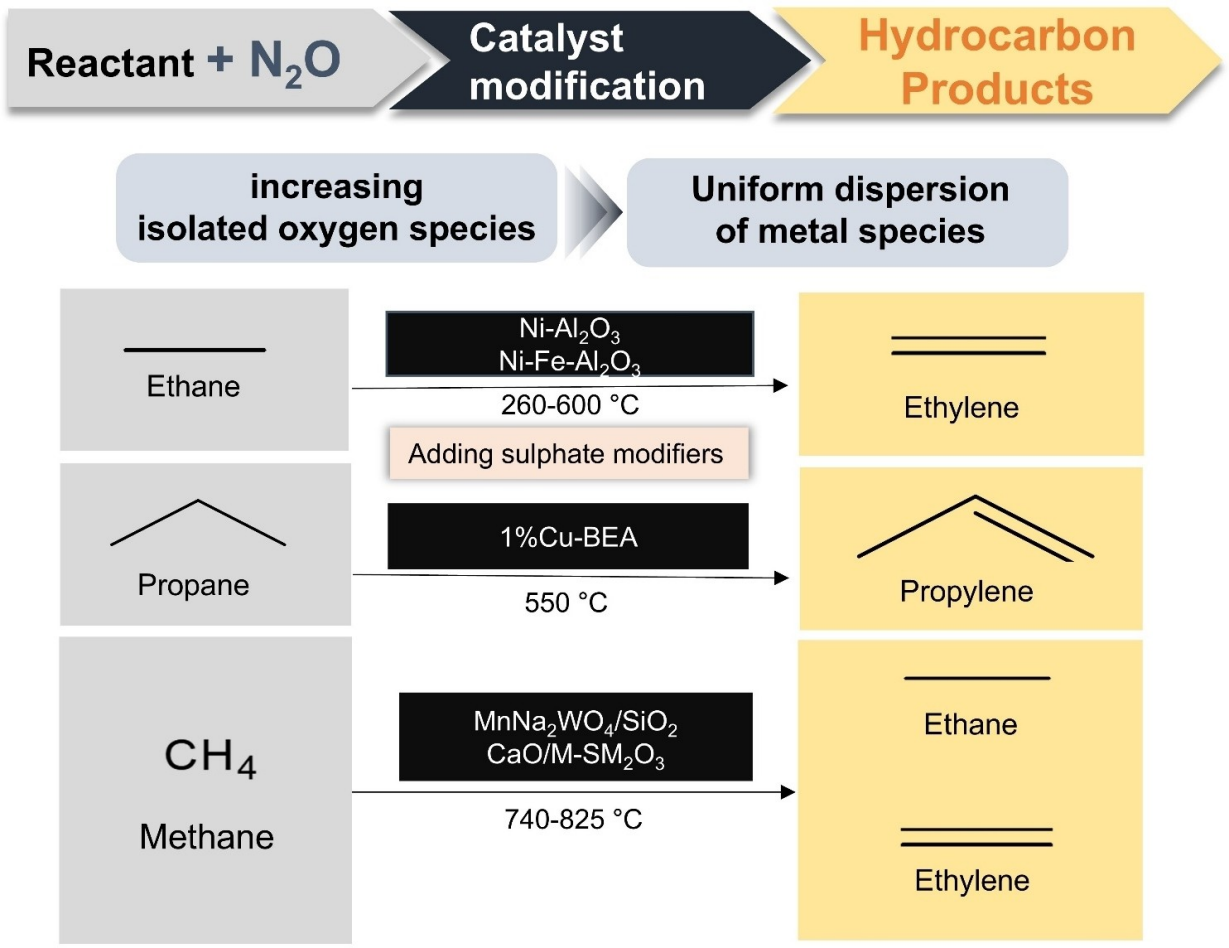
Schematic diagram of N₂O‐assisted hydrocarbon production.

The Beshara et al. used an inorganic support, h‐BN, to overcome the disadvantage that conventional metal oxide catalysts.^[84]^ It exhibits strong interaction with the substrate due to their redox activity, resulting in continuous oxidation, which reduces selectivity. As a method to control Fe speciation, ball milling was demonstrated to be the most ideal method to enable easy exfoliation of BN while inducing defects in the crystal lattice to create anchoring points for the metal species. The atomically distributed Fe species were stable for more than 1080 min. Figure 10 presents a summarized flowchart of N₂O‐assisted hydrocarbon production, illustrating the reactants, target products, catalysts, and key objectives of catalyst modification in this paper. Table 4 shows the characteristics of catalysts for conversion to hydrocarbons. Table 5 shows the catalytic performance of conversion to hydrocarbons.

## 5 Ring Compound Products

In this section, the production of styrene and phenol using N_2_O as an oxidising agent is discussed. Ring compounds such as styrene and phenol are obtained through oxidative dehydrogenation (ODH) reactions. Phenol production is traditionally produced industrially through the cumene process. An acid catalyst is used to alkylate benzene and propylene to produce cumene. The cumene is further oxidized to cumene hydroperoxide, which is then acid‐catalysed to phenol and acetone. The disadvantage of cumene process is that the ratio of phenol and acetone produced does not match up with the ratio of phenol and acetone consumed. Too much acetone is produced for the demand for acetone to be economical.^[85]^

**Figure 11:**
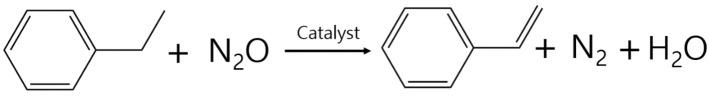
Oxidative dehydrogenation of ethylbenzene under N_2_O. Reprinted from Liu et al.,^[89]^ Copyright (2021), with permission from Elsevier.

The changing of benzene to phenol under N_2_O has attracted attention as a more economical and environmental alternative. Iron‐loaded zeolite catalysts have been identified as a promising catalyst for this reaction and have been studied until recently. The conversion of ethylbenzene to styrene in an N_2_O‐mediated environment has also been a topic of recent interest. Currently, commercial production of styrene is generated via the dehydrogenation of ethylbenzene. It is an endothermic and reversible reaction with potassium catalysts promoted by iron oxide catalysts.^[86]^ Superheated steam is used to mitigate carbon deposition and lead equilibrium transfer to the products through partial pressure reduction. The problem with this method is that the activation energy for the reaction is high (152 kJ/mol) and the energy consumption is extreme.^[87]^ The oxidative dehydrogenation of ethylbenzene (ODEB) is considered a more thermodynamically favourable method because it is endothermic and the conversion is not equilibrium limited.^[88]^ Under N_2_O conditions, ODEB follows the following reaction (Figure 11).

As a result of research on the N_2_O‐mediated reaction to form phenol from benzene, progress has been made in improving the rate of phenol formation. Nevertheless, the short lifespan of the catalyst due to rapid coking emerged as a major problem. Therefore, much research has been conducted on relieving catalyst coking. Meloni et al. revealed that catalyst deactivation mainly occurs during the decomposition and condensation of phenol to acid sites.^[90]^ Detailed catalyst deactivation properties, such as irreversible deactivation of the acidic sites at different time‐on‐stream (TOS) during the continuous reaction, have not been studied. Catalysts were manufactured by loading iron ions onto zeolites. The differences in catalyst structure and the effect of steam treatment were studied.^[91]^ A hierarchical Fe/ZSM‐5‐based catalyst (the Fe/ZSM‐5‐Hi2.8) synthesised with an optimised cetyltrimethylammonium bromide to an aluminium isopropoxide molar ratio of 2.8 showed great performances(phenol yield of 71.63 % after 5 min). This catalyst had the surface area of 315 m^2^/g. The Fe/ZSM‐5‐Hi2.8 catalyst showed a better performance than the microporous Fe/ZSM‐5 benzene oxidation. The two catalysts had similar Si/Al ratios, but the hierarchical Fe based catalyst had a smaller particle size. In addition, mesopores were formed more effectively on the hierarchical Fe catalyst, which had a combination of mesoporous and microporous structures that favoured the reaction. The micropore region was divided by the mesoporous matrix, allowing high accessibility to the Fe–O‐Al active sites. Moreover, the mesoporous structure could accommodate a lot of coke, thereby delaying the deactivation. The hierarchical Fe/ZSM‐5 had a lot of Fe–O‐Al sites and exhibited a stable crystal structure during regeneration. The phenol productivity catalysed by Fe/ZSM‐5‐Hi2.8‐F was 147.06 mmol_phenol_ g_cat_^−1^ for TOS of 1800 min. On the other hand, microporous Fe/ZSM‐5‐F had 53 mmol_phenol_ g_cat_^−1^ productivity for TOS of 420 min. Also, microporous Fe/ZSM‐5‐F had lower initial phenol formation rate compared with the hierarchical catalyst (12.41 *versus* 16.49 mmol_phenol_ g_cat_^−1^ h^−1^ for TOS of 420 min). Steam treatment enhanced the properties of the catalyst, which was favourable for the reaction. steam activation was performed at 650 °C, 1.9 mL/h of H_2_O and 1800 mL/h of He for 4 h. For the hierarchical catalyst without steam treatment, benzene conversion was 17.54 %; N_2_O conversion was 100 %, and the phenol yield was 32.88 %, which was considerably lower than the catalyst with steam treatment. This finding was due to the even distribution of fine Fe–O‐Al after steam treatment. The steam treatment migrated Al species and reacted with the Fe species extracted from the lattice to form a uniformly distributed fine Fe complex, which was primarily composed of additional ETRA framework Al species. Furthermore, the catalyst lifetime was prolonged about 330 % because of the steam treatment. This reaction was due to the formation of mesopores and a decrease in acidity. The steam treatment decreased the Brønsted and Lewis acidity of the catalyst, thereby reducing the rate of coke formation and producing a softer coke (Table 6, entries 1–4). After 10 regenerations, the catalyst shows only 81 % of the surface area and 65 % of the total pore volume of the fresh one. Al migration and aggregation occurred right after the first regeneration, indicating the irreversible deactivation of the acidic sites. Analysis also showed that regeneration did not completely remove the coke, and the coke progressively built up on the regenerated catalyst. After nine regeneration cycles, the N_2_O conversion was 100 %. On the contrary, benzene conversion increased from 13.74 % to 21.52 %, which showed an increase of the side reactions such as coking (Table 7, entries 1–2). In addition, the phenol yield decreased from 71.63 % to 36.63 %, indicating irreversible deactivation. After 10 regenerations, the initial phenol formation rate productivity on Fe/ZSM‐5‐Hi2.8 decreased to 13.22 mmol_phenol_ g_cat_^−1^ h^−1^ (ca. 20 % of decrease) and 76.86 mmol_phenol_ g_cat_^−1^ (17 % of decrease), respectively. As the reaction time increases, part of the soft coke followed reactions such as condensation at the acid site. Consequently, coke becomes graphitic, which required a high regeneration temperature. Therefore, a reaction time of 420 min or less is required to prevent this reaction.

**Table 6:** Characteristic of catalysts for conversion to ring compounds.

Entry	Catalyst	Method	Metal	Ration (wt.%)	Heat treatment	Sieve (mesh)	Brønsted (mmol/g)	Lewis (mmol/g)	Total (mmol/g)	Surface area (m^2^/g)	Micropore area (m^2^/g)	Mesopore area (m^2^/g)	Micropore volume (cm^3^/g)	Total pore volume (cm^3^/g)	Average pore diameter (nm)	Ref.
1	Hierarchical Fe/ZSM‐5‐Hi2.8	Hydrothermal	Iron(III) nitrate nonahydrate	Fe(1)	550 °C; 240–360 min	40–60	0.035	0.114	0.370	315	‐	‐	0.067	0.316	‐	[91]
2	Hierarchical Fe/ZSM‐5‐Hi2.8 (9 regeneration)	Hydrothermal	Iron(III) nitrate nonahydrate	Fe(1)	550 °C; 240–360 min	40–60	0.024	0.073	0.154	256	‐	‐	0.044	0.204	‐	
3	microporous Fe/ZSM‐5	Hydrothermal	Iron(III) nitrate nonahydrate	Fe(1)	550 °C; 240–360 min	40–60	0.022	0.113	0.183	302	‐	‐	‐	‐	‐	
4	Fe/ZSM‐5‐Hi2.8(no steam activation)	Hydrothermal	Iron(III) nitrate nonahydrate	Fe(1)	550 °C; for 240–360 min; steam activation (650 °C, 1.9 mL/h of H_2_O, 1800 mL/h of He, 240 min)	40–60	0.126	0.122	1.130	394	‐	‐	0.087	0.325	‐	
5	Fe/ZSM‐5‐ST	Ion exchange	Iron(III) nitrate	Fe(~1)	650 °C; 240 min; steam treatment (H_2_O/He, 1.9 mL/h/1800 mL/h, 650 °C, 240 min)	60–80	0.02	0.03	0.89^a^	196	104	92	0.1	0.28	‐	[92]
6	0.10Ce‐Co_2_ AlO_4_	One‐pot hydrothermal	Cerium(III) nitrate hexahydrate and Cobalt(II) nitrate hexahydrate and aluminium nitrate nonahydrate	Ce/Co=0.1	600 °C; 240 min	60–80	‐	‐	‐	64.9	‐	‐	‐	0.16	9	[89]
7	Ce‐Co/CNT	Hydrothermal	Cobalt(II) nitrate hexahydrate, Cerium(III) nitrate hexahydrate		500 °C; 240 min; N_2_	40–60			5628a.u.^b^	131	‐	‐	‐	0.27	1	[98]
8	K/Co_2_AlO_4_	One‐pot hydrothermal	Cobalt(II) nitrate hexahydrate, aluminium nitrate hydrates, Potassium carbonate	K(1.7)and Co(28.4)	600 °C; 240 min	40–60	‐			44.6	‐	‐	‐	0.13	‐	[100]
9	Co_2_AlO_4_	One‐pot hydrothermal	Cobalt(II) nitrate hexahydrate aluminium nitrate hydrates	‐	600 °C; 240 min	40–60	‐			44.9	‐	‐	‐	0.13	‐	
10	0.3Ce−7Co/OMA	Hydrothermal method	Co(NO_3_)_2_0.6 H_2_O, Ce(NO_3_)_3_0.6 H_2_O,	Ce(0.3) and Co(7)	600 °C; 240 min	40–60			0.645^c^	257.8				0.35	4.65	[101]
11	Cr‐5Si‐Al‐850	EISA method	Ethyl silicate and 2.746g Cr(NO_3_)_3_⋅9H_2_O		850 °C; 240 min				0.12^d^	156.2				0.29	6.6	[102]

^a^week (0.52 mmol/g), strong (0.37 mmol/g), ^b^medium strong (1525 a.u.), strong (4103 a.u.), ^c^moderate (0.271 mmol/g), Strong(0.374 mmol/g), ^d^moderate(0.021 mmol/g), Strong(0.099 mmol/g).

**Table 7:** Catalytic performance of conversion to ring compounds.

Entry	Catalyst			Reaction condition					Reactant conversion			Product selectivity					Yield	Productivity	Coke amount (time)	Ref.
	Name	Loading	Size (mesh)	Temperature(°C)	pressure	Atmosphere	Space velocity	time	N_2_O	Ethylbenzene (%)	benzene	styrene	toluene	benzene	phenol	styrene	phenol	phenol		
1	Hierarchical Fe/ZSM‐5‐Hi2.8	2 g	40–60	425	‐	N_2_O:Benzene=1 : 10	4160 mL/g· h	~1800 min	100	‐	13.74	‐	‐	‐	60–95	‐	71.63	92.88 mmol_phenol_/g_catalyst_	1.5	[91]
2	Hierarchical Fe/ZSM‐5‐Hi2.8(9 regeneration)	2 g	40–60	425	‐	N_2_O:Benzene=1 : 10	4160 mL/g· h	~1800 min	100	‐	21.52	‐	‐	‐	50–75	‐	36.63	76.86	~3.9	
3	microporous Fe/ZSM‐5	2 g	40–60	425	‐	N_2_O:Benzene=1 : 10	4160 mL/g· h	~1800 min	100	‐	17.54	‐	‐	‐	30–70	‐	74.89	53.13	1.1	
4	Fe/ZSM‐5‐Hi2.8(no steam activation)	2 g	40–60	425	‐	N_2_O:Benzene=1 : 10	4160 mL/g· h	~1800 min	100	‐	9.88	‐	‐	‐	‐	‐	32.88	12.98	3.1	
5	Fe/ZSM‐5‐ST	2 g	‐	425	0.101 MPa	N_2_O:Benzene=1 : 10	4320 mL g_catalyst_^−1^ h^−1^	~3 h	78.8–100	‐	‐	‐	‐	‐	52–78.8	‐	45–80	‐	‐	[92]
6	0.10Ce‐Co_2_AlO_4_	1 g	40–60	500	‐	N_2_O:Ethylbenzene=3 : 1	9000 h^−1^	1 h	‐	61.5	‐	80.6	0.8	4	‐	48.8	‐	‐	1.2 % (6 h)	[89]
7	0.10Ce‐20wt %Co/CNTs	0.5 g	‐	500	‐	N_2_O:Ethylbenzene=3 : 1	9000 h^−1^	1 h	‐	41	‐	84	trace	ab. 3	‐	ab. 35	‐	‐	‐	[98]
8	Ce‐7Cr‐mAl_2_O_3_	1.5 g	40–60	500	Atmospheric	N_2_O:Ethylbenzene=3 : 1	6000 h^−1^	4 h	100	68	‐	80	‐	‐	‐	54	‐	‐	‐	[99]
9	Co_2_AlO_4_	1 g	40–60	500	Atmospheric	N_2_O:ethylbenzene=3 : 1	‐	‐	80–90	44.3	‐	70.1	0.3	2.4	‐	‐	‐	‐	‐	[100]
10	K/Co_2_AlO_4_	1 g	40–60	500	Atmospheric	N_2_O;ethylbenzene=3 : 1	‐	‐	100	62	‐	85.1	2.3	1.3	‐	‐	‐	‐	‐	
11	0.3Ce− 7Co/OMA	1 g	40–60	500		N_2_O:ethylbenzene=3 : 1		~24 h	80	52.1		88.7				46.2			3.52 %/h	[101]
12	Cr‐5Si‐Al‐850	1.5 g	40–60	500	Atmospheric	N_2_O:ethylbenzene=3 : 1		4 h		70		62				44				[102]

Since Fe‐ZSM‐5 was discovered for the nitrogen dioxide‐mediated changing of benzene to phenol, the role of Fe species in the catalyst and the effect of catalyst pore size have been studied. But attention has been concentrated on the role of Fe ions and mesopores, and few attempts have been made to study the acidity change with post‐treatment. Ouyang et al. conducted an exploration of acids before and after steam treatment to enhance the benzene oxidation performance of the H‐ZSM‐5 catalyst to phenol.^[92]^ The reaction was carried out at 425 °C. The catalysts that were steam treated at high temperatures had Al removed from the zeolite framework, and the crystal size decreased. It also reduced the micropore. After steam treatment of Fe‐ZSM‐5, it was reduced from 244 to 104 m^2^/g (Table 6, entry 5). Therefore, the proportion of mesopores increased. Mesopore provided the diffusion path for the reaction interface and mass transfer. This led on high initial selectivity. The initial phenol selectivity of Fe/ZSM‐5‐ST (with steam treatment) was 78.8 %, which was higher and more stable than Fe/ZSM‐5 (without steam treatment). The steam‐treated catalyst showed relatively high stability and initial selectivity, but the selectivity considerably decreased after 90 min. The Fe/ZSM‐5‐ST catalyst showed lower N_2_O conversion than the FE/ZSM‐5 catalyst. It initially maintained a value of 100.0 % and then decreased rapidly to 78.8 %. With regard to the yield, FE/ZSM‐5‐ST achieved a higher initial yield of 80 %, indicating that fewer acidic sites were favourable for selectivity and stability. Steam treatment induced changes in Fe species. The reduction of the aggregated Fe_2_O_3_ nanoparticles resulted in an a lot of number of isolated Fe ions in tetrahedral and oligomeric Fe(III)_*x*_O_*y*_. Steam treatment had fewer acidic sites because of the de‐aluminisation of the zeolite. In particular, the initial Brønsted acid sites were almost entirely lost. Moreover, cluster of the extra‐framework Al resulted in drop of Lewis acidity after steaming treatment. Furthermore, after steaming, Fe/ZSM‐5‐ST had a small amount of Brønsted acid and a certain amount of Lewis acid. Notably, steaming had a stronger effect on Brønsted acid than Lewis acid, thereby reducing the Brønsted site/Lewis acid site. The strong acidic site of Fe/ZSM‐5‐ST after steam treatment was 0.37 mmol/g. It was less than that before steam treatment (1.05 mmol/g), and the weakly acidic site was 0.52 mmol/g, which was less than that before treatment (1.33 mmol/g) (Table 6, entry 5). In brief, the low acidity (low Brønsted/Lewis ratio) and high mesopore ratio by steam treatment increased the catalyst lifetime and initial selectivity of phenol. Fe/ZSM‐5‐ST with a Brønsted/Lewis ratio of 0.67 showed the highest performance. The authors suggested that ion exchange and water vapour treatment could be utilised to control catalyst acidity through this experiment.

The N_2_O‐assisted ODEB catalyst has been reported as transition metal oxides,[^93^, ^94^, ^95^] acid catalysts like alumina, zeolites and phosphates.[^96^, ^97^] However, their selectivity and stability for styrene are not sufficient. research attempts have been ongoing to find catalysts that result in high selectivity and conversion. Liu group presented a Ce–Co–Al mixed oxide synthesized via a one‐pot hydrothermal process as a high‐performance catalyst.^[89]^ The Ce‐Co_2_AlO_4_ catalyst (0.1Ce‐Co_2_AlO_4_) synthesised with a Ce/Co ratio of 0.1 showed the best ethylbenzene conversion and styrene selectivity. Ethylbenzene conversion was as high as 60.5 %, and styrene selectivity was as high as 80.6 % (1 h TOS, 500 °C, GHSV 9000 h^−1^). The styrene yield reached 48.8 %. This result was superior to other literature that has studied the catalytic dehydrogenation of ethylbenzene.

**Figure 12:**
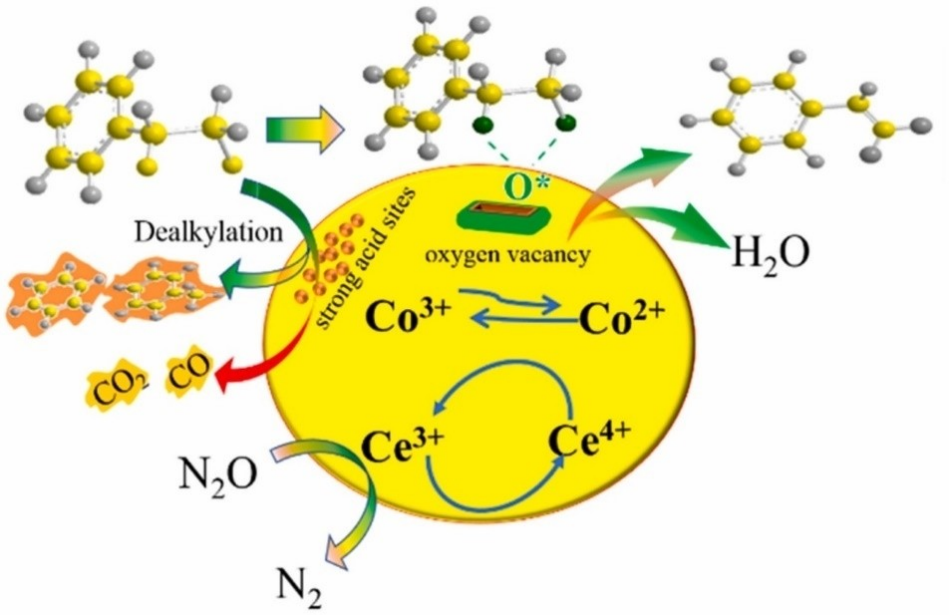
Proposed reaction for ODEB assisted by N_2_O over the 0.10 Ce‐Co_2_AlO_4_ catalyst. Reprinted from Liu et al.,^[89]^ Copyright (2021), with permission from Elsevier.

Figure 12 is the reaction pathway proposed by the Liu group. Active oxygen species formed styrene from ethylbenzene through dehydrogenation of the ethyl group. As a result, oxygen vacancy was generated, and Co^3+^/Ce^4+^ was reduced to Co^2+^/Ce^4+^. Due to the excellent oxygen release and storage capacity of cerium oxide, oxygen vacancy was restored, and Co^3+^/Ce^3+^ was generated. Then, N_2_O decomposes to generate active oxygen species, and Co^3+^/Ce^4+^ was achieved. The increase of Ce/Co ratio had a good effect on the enhancement of surface area, pore volume and mobile surface oxygen species. The Ce/Co ratio was proportional to the surface area and pore volume because of the high dispersion and porous nature of CeO_2_ particles. The interaction between CeO_2_ and Co_2_AlO_4_ caused a decrease in particle size, which increased the number of active sites exposed to the catalyst surface, thereby enhancing the catalytic performance. The addition of Ce improved the surface area compared with the surface area of Co_2_AlO_4_ (44.9 m^2^/g) (Table 6, entry 6). The addition of Ce promoted the reduction of Co surface oxygen, which reduced the catalytic reduction temperature. Therefore, the number of mobile surface oxygen species increased. The interaction with Ce weakens the Co–O bond. Ce loading was proportional to the amount of Ce^3+^ and Co^3+^, indicating that the re‐oxidation reaction of Co^2+^ (Ce^4+^ + Co^2+^→Ce ^3+^ + Co^3+^) occurred. In addition, the higher the Ce/Co ratio, the faster the oxygen exchange of cerium, and the more oxygen vacancies occurred during the process, which affected the distribution of O species. This trend led to an increase in the number of O alpha, which played an important role in oxidation with high mobility. The coke formed on the catalyst with a Ce/Co ratio of 0.1 (0.1Ce/Co_2_AlO_4_) was the most unstable. Moreover, the amount of coke on 0.1Ce/Co_2_AlO_4_ was the least. In brief, the Ce/Co ratio of 0.1 improved resistance to coke. This was advantageous for the reversibility of mobile oxygen, resulting in the highest conversion of ethylbenzene. The addition of Ce changed the distribution of acid sites. Strong acid sites decreased with the increase of Ce/Co ratio by masking the surface strong acid sites with Ce oxide. Therefore, Ce loading increases the number of moderate acidic sites, which were favourable for dehydrogenation, thereby increasing the styrene selectivity.

Liu et al. presented Co‐Ce/CNTs with carbon nanotubes as support, which were the most active and stable carbon nanomaterials due to their physical structure and surface properties.^[98]^ Ce‐Co/CNTs were prepared by loading Ce–Co onto carbon nanotubes via a hydrothermal method for ethylbenzene dehydrogenation to produce styrene. Both samples had the same surface area and pore volume for Co added with carbon nano tube (Co/CNTs) and co‐loading of Ce and Co (Ce‐Co/CNTs). However, the co‐loading of Ce and Co showed better catalytic performance, indicating that the surface area was not the main factor determining catalytic performance. In Ce‐Co/CNT catalysts, Co_3_O_4_ and CeO_2_ forms were found. When Ce was loaded onto CNTs with Co, the following effects were observed. First, the interaction between the Co and Ce species reduced the crystal size of Co_3_O_4_, which improved its thermal stability. Second, the interaction of Ce with Co or CNTs weakened the Ce–O bonds, resulting in the generation of more active oxygen species. The O_alpha_/O_total_ ratio of 0.1Ce‐20wt %Co/CNTs was 67.34 %, which was the highest among the prepared samples. 0.1Ce‐20wt %Co/CNT showed the ethylbenzene conversion of 41 % and styrene selectivity of 84 % were achieved in 60 min, and the maximum yield was 35 %.

Mesoporous ceria‐chromium‐alumina oxide (Ce‐7Cr‐mAl_2_O_3_) was utilized for the production of styrene at 500 °C.^[99]^ The catalyst synthesized by loading only Cr with 7 % content (7Cr‐mAl_2_O_3_) showed better performance than other ratios. When using 7Cr‐Al_2_O_3_, the ethylbenzene conversion increased by 44 % compared with Al_2_O_3_, reaching 67 %. Styrene selectivity was also high at 75 %, resulting in a high styrene yield of 50 %. When Ce was added (Ce‐7Cr‐mAl_2_O_3_), the process became more efficient because of the synergistic effect between Ce and Cr, resulting in a higher styrene yield of 54 %. Among the oxygen species, the proportion of alpha oxygen species was the highest in Ce‐7Cr‐mAl_2_O_3_ at 60.05 %. When Ce and Cr were loaded, more oxygen vacancies occur because of the charge balance among the elements, which contributed to the high alpha oxygen content in Ce‐7Cr‐mAl_2_O_3_. Ce‐7Cr‐mAl_2_O_3_ (Cr/Al=7, Ce/Cr=0.1, molar ratio) showed the highest catalytic activity but a lower surface area of 172.3 m^2^/g compared with mAl_2_O_3_ or Cr‐mAl_2_O_3_.This result indicated that the performance activation by Ce‐7Cr‐mAl_2_O_3_ was dependent on chemical factors. The addition of Cr promoted the decomposition of N_2_O by making the Cr–O bond weak because of the strong bonding of Cr oxide and support and activated the oxidation of ethylbenzene by promoting the formation of active oxygen. It also enhanced the adsorption of N_2_O, which facilitated the reaction. The presence of Cr species activates N_2_O conversion, reaching 100 % N_2_O conversion in samples with more than 7 % Cr content (including Ce‐7Cr‐mAl_2_O_3_). As the Cr content increased from 5 % to 10 %, more moderately acidic sites and fewer strongly acidic sites were observed, which were favorable for ethylbenzene dehydrogenation. On the contrary, when the Cr content was 15 %, the strongly acidic sites increased, resulting in a decrease in catalytic activity. The addition of Ce increased the moderate acidic sites by 0.13 mmol/g, reaching 0.32 mmol/g compared with 7Cr‐mAl_2_O_3_. At a low Cr content (5–7 %), the Cr^6+^ content increased to 52.36–53.56 %. When the Cr content exceeded 10 %, the proportion of Cr^6+^ decreased, and Cr^3+^ became the dominant species. The presence of more active sites in the Cr^6+^ oxide was advantageous in the dehydrogenation reaction of ethylbenzene. The addition of Ce improved the catalyst reducibility. The addition of Cr–Ce did not change the morphology of the catalyst but caused a small number of particles to expand and sinter. Therefore, the particle size of Ce‐7Cr‐mAl_2_O_3_ was 13 nm, which was larger than other samples. In addition, Cr and Ce intensify the coke formation. This tendency was proportional to the loading amount of Cr. However, the stability of the deposited carbon decreased with the addition of Ce and Cr. The carbon formed on the catalyst surface was oxidized to generate oxide‐containing groups, creating new active sites for ODH reaction. Therefore, coking can act as a positive factor. When ethylbenzene was added, N_2_O conversion increased to 30 %. This reaction indicated the combined effect of N_2_O decomposition and dehydrogenation of ethylbenzene, which was explained through the catalytic reaction cycle (**Eqs. 1–4**). [ ] in the equations indicates an oxygen deficit. The dehydrogenation of ethylbenzene consumes oxygen species to create an oxygen deficit (**Eq. 1**), which was recovered by the decomposition of N_2_O (**Eq. 2**). In particular, cerium oxide acted as a cross‐linking agent to donate oxygen atoms to the oxygen deficit because of its excellent oxygen storage/release ability (**Eq. 3**). Then, the remaining oxygen deficit was recovered by N_2_O (**Eq. 4**).
(1)

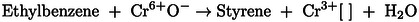



(2)

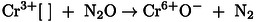



(3)

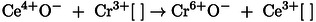



(4)

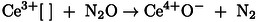




The generation of styrene from ethylbenzene over Co–Al spinel oxides and K‐modified catalysts showed high performance.^[100]^ When the molar ratio of N_2_O/ethylbenzene was 3, the maximum initial ethylbenzene conversion of 44.3 % and styrene selectivity of 70.1 % were achieved when the molar ratio of Co/Al was 2 (Co_2_AlO_4_) and when only Co species was introduced at 500 °C. Further increasing the molar ratio of N_2_O/ethylbenzene or the addition of Co increased the ethylbenzene conversion but decreased the selectivity, resulting in a decrease in yield. In brief, more benzene and smaller hydrocarbon molecules were produced. Notably, the K‐modified Co_2_AlO_4_ showed a higher ethylbenzene conversion of 62.0 %. Moreover, the styrene selectivity was high at 85.1 %. The conversion of N2O was also increased from about 80–90 % in Co_2_AlO_4_ to almost 100 % after K‐modification. The complete decomposition of N_2_O provided sufficient reactive oxygen species for the dehydrogenation of ethylbenzene. As the molar ratio of Co/Al increased, the surface area of the catalyst decreased because of the increase in texture density, but the addition of potassium (K) species had little effect on the surface area and pore structure. When the molar ratio of Co/Al was higher than 2, a small amount of coke was generated, which increased the surface area. Interestingly, K/Co_2_AlO_4_ showed little coke deposition as the surface area and pore structure remained almost unchanged after the reaction. This phenomenon could be explained by the high catalytic activity of the K/Co_2_AlO_4_ catalyst. The addition of Co to Al_2_O_3_ resulted in a Co–Al structure and increased catalyst crystallinity. In this case, the molar ratio of Co/Al and the crystal size were proportional. With the addition of Co, sharp tips were observed as blunt particles. When K was further loaded, K/Co_2_AlO_4_ showed the same spinel structure, and the low content of K on the spinel surface showed high dispersion. No difference in morphology was observed after K doping. The C‐C bond of ethylbenzene plays a role in dealkylation and ring opening reactions that caused the degradation of Al_2_O_3_ by strong acid sites. The addition of Co reduced the total amount of acid spots, and the degree of reduction was large as the loading amount increases. K‐modification weakened strong acids because of the neutralising effect of alkaline K‐oxide. This reaction resulted in high styrene selectivity because of the reduced dealkylation and ring opening reactions. It also prevented carbon deposition onto the catalyst surface and oxidised the coke to CO_2_/CO molecules. Consequently, K/Co_2_AlO_4_ exhibited the smallest coke formation, and the coke was less stable, thereby preventing the irreversible deactivation of the active site. The addition of K to Co‐oxide weakened the bond of Co^3+^–O– and facilitated its reduction to CO^2+^ (2Co^3+^–O–→Co^3+^–O‐O–Co^3+^→O_2_ + 2Co^2+^), which accelerated the generation of active oxygen species and promoted the dehydrogenation reaction. The doping of Co–Al oxide with K promoted the electron transfer from K to Co species by the electron donor effect of K, resulting in more Co^2+^ in K/Co_2_AlO_4_, which promoted the decomposition of N_2_O. As the molar ratio of Co/Al increases, the value of Co^3+^/Co^2+^ increases, thereby promoting the dehydrogenation reaction of ethylbenzene. By contrast, when the molar ratio of Co/Al was 3, the conversion rate decreased because of the low surface acid content. As the content of Co species increased, the alpha oxygen content also increased. Therefore, the addition of potassium resulted in the largest alpha oxygen content (O_alpha_/O=48.36 %). O_alpha_ was considered as the active species in oxidation reactions because of its high mobility. The mobile oxygen oxidised the coke to CO_2_/CO molecules and promoted dehydrogenation. This process explained the high ethylbenzene conversion and small amount of coke deposition on the K/Co_2_AlO_4_ catalyst.

**Figure 13:**
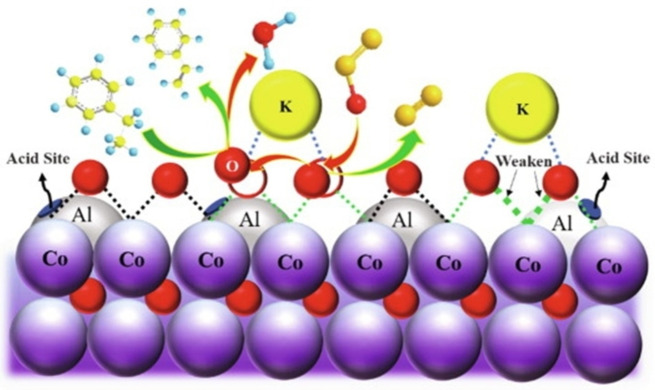
Conversion of ethylbenzene to styrene under N_2_O with a K/Co_2_AlO_4_ catalyst. Reprinted from Liu et al,.^[100]^ Copyright (2022), with permission from the Korean Society of Industrial and Engineering Chemistry.

Figure 13 shows the reaction mechanism of the ODH of ethylbenzene. Equation 5 and 6 indicate the reaction pathways. [ ] represents oxygen vacancy. After ethylbenzene adsorbed on the catalyst, it created an oxygen vacancy by giving electron from an oxygen atom to the Co^3+^ species. The H‐H released in this process reacted with surface oxygen to form H_2_O (**Eq. 5**). Subsequently, Co^2+^ was activated by N_2_O to produce Co^3+^–O^−^ species and N_2_ to fill the oxygen deficit (**Eq. 6**).
(5)

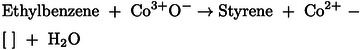



(6)

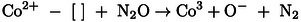




The Al_2_O_3_ material presented ordered mesoporous structure and good physical and chemical properties, was employed as a support to prepare the CeO_2_–Co_3_O_4_ binary oxide catalysts for the possible use in the N_2_O‐assisted Ethylbenzene dehydrogenation.^[101]^ In particular, the analysis related to the precipitation of cokes was carried out. The 0.3Ce‐7Co/OMA (Order mesoporous alumina) yielded a high initial ethylbenzene conversion of 36.8 %. It took 240 min to reach steady state and finally showed a stable ethylbenzene conversion of 52.1 %. The styrene selectivity was 88.6 %, and the N_2_O conversion was about 80 %. The styrene yield reached 46.2 %. The C‐C bond of hydrocarbon formed decomposition products at strongly acidic sites, resulting in carbon deposition. The oxygen‐containing functional groups (carbonyl, quinone) abundant in the carbon deposits provided moderate acid sites to activate the dehydrogenation reaction. As the reaction progressed, the carbon formed gradually accumulated on the catalyst surface until the strong acid sites were completely covered with precipitated carbon in about 960 min. In other words, the strong acid was a factor that alleviated the carbon accumulation on the catalyst surface to reach a balanced state and exhibit stable catalytic activity. After regeneration in air at 600 °C for 240 min, the initial ethylbenzene conversion rate showed little difference from the fresh catalyst. All results indicate that the 0.3Ce‐7Co/OMA catalyst has good stability, and its applicability can be further developed.

**Figure 14:**
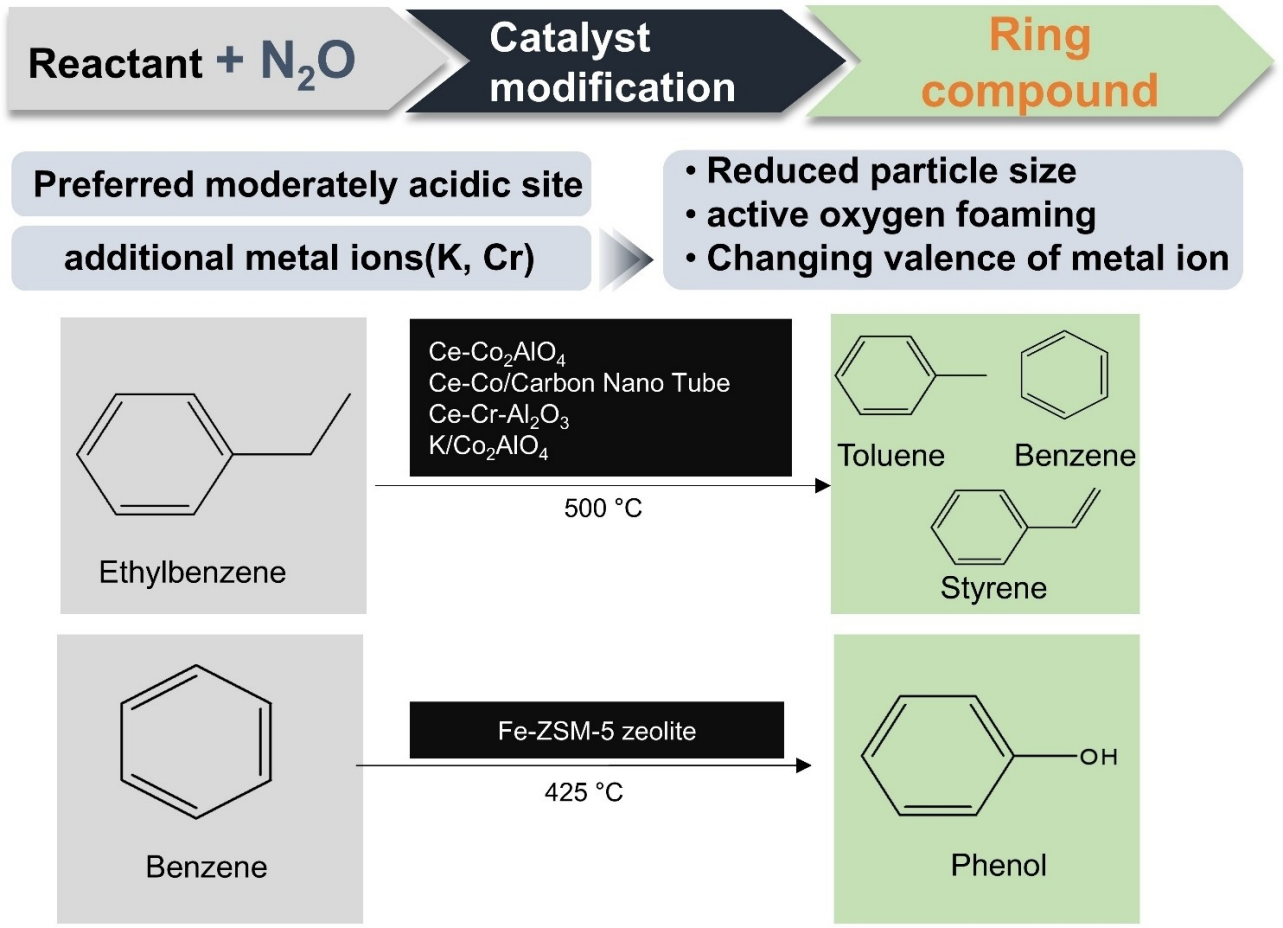
Schematic diagram of N₂O‐assisted ring compound production.

Thermally treated SiO_2_‐promoted Al_2_O_3_ was prepared to optimize surface properties through interaction between Si and Al.^[102]^ The promotional effect of SiO_2_ modification on the catalytic performance of N_2_O‐ODEB was systematically investigated. The relationship between the interaction of SiO_2_–Al_2_O_3_ and catalytic performance were studied. Cr‐5Si‐Al‐850 showed the best catalytic performance. The styrene selectivity was 62 %, the ethylbenzene conversion rate was about 70 %, and the styrene yield was nearly 44 %. In contrast, the Cr‐Al‐850(without Si) showed lower yields, with a styrene selectivity of 57 % and a styrene yield of about 40.5 %. The catalyst calcination temperature ranged from 700 to 900 °C, with 850 °C showing the best performance. The Cr‐Si‐Al‐850 catalyst showed the largest specific surface area with the smallest pore size. The large specific surface area provides additional active sites, which improves catalytic performance. As the calcination temperature increased, the channels became more distinguishable and uniform, making it easier for reactants to diffuse and access the active sites. In addition, with the increase of calcination, the strong acid sites were reduced, and the ratio of Cr^6+^/(Cr^6+^+Cr^3+^) was gradually increased by interspecies interactions. Cr species with higher valence states contribute to high catalytic performance. In addition, the ratio of O‐alpha/O_alpha_+O_beta_+O_gamma_ increased to 46.49 % with the increase of calcination temperature. This indicates that the interaction between Si and Al species can induce additional surface vacancies to promote oxygen activation. The ratio of O_alpha_ to Cr^6+^ was maximum in Cr‐5Si‐Al‐850. In the Cr‐5Si‐Al‐900 catalyst, the catalytic performance was degraded because the changed nature of the catalyst at the elevated calcination temperature failed to generate an adequate number of active sites, resulting in poor N_2_O decomposition and unfavourable adsorption of ethylbenzene. On the other hand, Cr‐5Si‐Al‐850 showed improved adsorption of ethylbenzene, which played an important role in improving the catalytic performance. Figure 14 presents a summarized flowchart of N₂O‐assisted ring compound production, illustrating the reactants, target products, catalysts, and key objectives of catalyst modification in this paper. Table 6 shows the characteristics of catalysts for conversion to ring compounds. Table 7 shows the catalytic performance of conversion to ring compounds.

## 6 Summary

This review deals with N_2_O‐assisted oxidation processes. N_2_O‐assisted oxidation uses N_2_O as an oxidant to selectively oxidise reactants to obtain the desired high‐value materials. The most challenging aspect of the process is how to prevent deep oxidation. Deep oxidation is undesirable because it results in lower yields of the target product and higher production of carbon oxides such as CO_2_. The products that can be derived from selective oxidation reactions can be broadly categorised as oxygenates, hydrocarbons (including hydrogen) and ring compounds. Figure 9 shows a simple schematic of the reactions covered in this paper along with the experimental conditions.

**Oxygenates product**: The oxygenates that can be produced are methanol, DME and other carbonyl compounds such as formaldehyde. In the selective oxidation of methane, an increase in temperature increases the conversion rate. On the contrary, above a certain level of conversion, deep oxidation is activated, which increases the selectivity of carbon oxides such as CO and CO_2_ and decreases the selectivity of high‐value‐added substances.

Catalyst activity has been improved by changing the preparation method, altering the metal active site structure and species distribution, and changing the surface oxygen species stability. In common, the improvement of catalysts for the selective oxidation of methane generated reactive oxygen species, increased their stability, and increased the tendency of reactants attached to iron species to migrate to silanol groups. This method prevents deep oxidation and increases the selectivity of high‐value products (methanol, DME, formaldehyde).

The metal used was mostly iron and the support was mainly zeolite. The high amount of alumina in the zeolite frame can favour the stabilization of metal species and the creation of active sites. The reduction of iron oxide on the catalyst surface, high metal dispersion, in turn, led to high catalytic activity. The metal species distribution is a factor that affects the surface oxygen species generation. The amount of metal loading and pH are factors that affect the amount and distribution of acid sites.

Increased methane conversion requires increased N_2_O efficiency, increased oxygen species efficiency, and increased reactant diffusion. Brønsted acid catalyses the dehydration of methanol to produce DME or promote further oxidation. In common, having wide pores resulted in high catalytic activity. This is due to the improved accessibility of reactants to the pores where the active sites are located.

There have been some attempts to prepare bimetallic catalysts with Cu. When copper‐loaded catalysts are used, the low water partial pressure increases the rate of methanol formation, aids in methanol desorption, and generates of active sites.

**Hydrocarbon product**: Recent attempts have been made to control metal species such as identifying the effect of reduction temperature, preparing the LDH form, and modifying the metal active site. This improved the dispersion of metal species uniformly which showed high activity by increasing the isolated oxygen species. The phase of the metal species was explored with forms favourable for reactant decomposition (adsorption) and forms unfavourable for deep oxidation.

In addition, the catalyst surface properties were controlled so that the hydrogen generated from dehydrogenation could be consumed by the oxygen species generated by the decomposition of N_2_O. A high surface area is a desirable morphology that increases the adsorption of oxygen species. Selectivity was improved with good mobility of active oxygen species, and a high number of isolated oxygen species favoured the reaction and prevented deep oxidation. It was found that the addition of appropriate modifiers can be advantageous for oxygen species control. The role of the modifier is to modify the morphology of the metal species to increase the isolated oxygen species. Isolated oxygen species have positive impact on ethylene production and desorption. The use of water changed the bimetallic oxygen, which causes deep oxidation, to monotonic, resulting in high methane conversion and C_2_ selectivity.

Many papers have demonstrated the effectiveness of introducing N_2_O over other oxidants (O_2_). N_2_O improved the target material selectivity by removing oxygen species that cause deep oxidation. In addition, N_2_O increases the separation of lattice oxygen species by re‐oxidizing the reduced catalyst slower than oxygen. N_2_O also contributed to the improvement of C_2_ selectivity due to its superior ability to deliver oxygen species compared to carbon dioxide.

**Ring compound**: The conversion of benzene to phenol has recently been studied for the reduction of coking and the effectiveness of post‐treatment to change acidity has been reported. By reducing the micropores and widening the mesopores, it was possible to delay deactivation as well as improve the accessibility favourable for diffusion and mass transfer. It was achieved by steam treatment.

The reaction to form styrene from ethylbenzene is a relatively new concept compared to other reactions, so increasing the selectivity and conversion is the most urgent step. The catalysts are mainly Ce and Co, and the addition of additional metal ions such as K and Cr has been proposed. The effect of co‐loading is to reduce the particle size to secure thermal stability and increase the active site. Co loading weakens the bonding of metal and oxygen and increases the formation rate of highly mobile active oxygen, i. e., alpha‐site. This has the effect of increasing the oxidation of ethylbenzene and improving the adsorption of N_2_O. In addition to this, it also improves the dispersion and aids in electron transfer to activate the reaction. Furthermore, metal co‐loading modifies the electron valence of metal ions on the catalyst surface to improve performance.

In the conversion of ethylbenzene to styrene, it is reported that surface area has little effect on catalyst performance. Nevertheless, a large surface area can provide additional active sites and increasing the uniformity and accessibility of the pores by high calcination temperatures can also be a performance improvement point. Changing in oxygen species is primary research issue on the production of styrene from ethylbenzene. By optimizing the addition ratio during the co‐loading of the metal, it is possible to generate alpha oxygen by rapid oxygen exchange leading to many oxygen deficiencies. Depending on the manufacturing variables (including calcination temperature, etc.), the interaction between the two loading metals can be favoured in the direction of further oxygen activation.

During the production of ring‐coupled products, the control of acid point is an important factor that is directly related to coking, i. e. the stability of the catalyst. In general, strongly acidic sites are avoided and moderately acidic sites are preferred. Strong acids can induce ring opening reactions and reduce the yield of the ring compound. In the process of benzene conversion to phenol, reducing the acidity is suggested to increase the life of the catalyst. Therefore, reducing the acid sites by de‐alumination, such as steam treatment, has been suggested as a method. It has been found that too long reaction time makes it difficult to regenerate coke, which means that the reaction time can be a factor in coking control. On the other hand, in the process of converting ethylbenzene to styrene, coke was presented as a positive factor. It was suggested that the oxygen‐containing functional groups of carbon deposits accumulated in strong acids can act as intermediate acid points that can activate the dehydrogenation reaction.

## 7 Conclusions and Perspectives

This review highlights the advancements in N₂O‐assisted selective oxidation processes, which utilize N₂O as an oxidant to selectively convert abundant feedstocks, such as methane, ethane, and benzene, into high‐value products like methanol, dimethyl ether (DME), and styrene. Significant progress has been achieved through catalyst modifications, including optimizing metal loading, active site distribution, and pore structure. These advancements have enhanced the stability of reactive oxygen species and improved the interaction between reactants and active sites, effectively mitigating deep oxidation and increasing the yield of target products.

Despite these achievements, several challenges remain that hinder the commercialization of this process. One important limitation is that carbon dioxide, especially CO_2_, is produced as a byproduct due to deep oxidation. Future research should prioritize catalyst designs that minimize active oxygen recombination and reduce the retention time of reactants at active sites. It is essential to improve the N₂O conversion efficiency without causing excessive side reactions. This strategy can also improve the currently lacking yield achievable. In order to balance the conversion and selectivity, the catalyst should be designed for the specific reaction and target product.

Another important limitation is the irreversible deactivation of the catalyst. As mentioned by Ouyang group,^[91]^ if the catalyst experiences irreversible deactivation, it may not be regenerable or may recover slowly even if thermal energy is applied. This limitation can greatly interfere with the operation of the process. In particular, it is important to minimize irreversible deactivation because the catalyst plays a very important role as an initiator of the reaction by decomposing N_2_O and reactants. In heterogeneous catalysis using N_2_O as an oxidizing agent, catalyst deactivation occurs due to both the of N_2_O itself and the reaction conditions. However, their relative importance depends on the specific catalyst and conditions. First, regarding the effect of N_2_O: Catalyst deactivation can occur if the oxygen species derived from N_2_O decomposition remain adsorbed on the active site, preventing further catalytic activity. Oxygen species from decomposition of N_2_O were strongly adsorbed, covered the oxygen deficiencies on the surface, and poisoned the catalyst surface.^[103]^ Additionally, catalysts with unstable redox properties (e. g., Fe_3_O_4_ and Mn_3_O_4_‐based materials) may undergo overoxidation in an N_2_O‐rich environment, shifting to an inactive oxidation state.^[104]^ Second, regarding reaction‐induced deactivation: In contrast to N_2_O‐related deactivation, reaction conditions also contribute significantly. Coexisting gases such as H_2_O can cause poisoning through competitive adsorption,^[57]^ and byproducts can condense on active sites, leading to deactivation.^[90]^ Additionally, prolonged reaction times can lead to coke formation, which, if converted into graphite‐like structures, becomes challenging to remove and regenerates less effectively.^[91]^ Regarding the comparison with stronger oxidants, deactivation can be worse with more reactive oxidants. For example, O_2_ which is stronger and more conventional oxidant compare with N_2_O, is often associated with greater deactivation than N_2_O due to its higher reactivity, which promotes the formation of non‐selective oxygen species. This can lead to increased local heat production and hot spots, particularly in OCM, accelerating catalyst degradation.^[76]^ The Asami group compared different oxidants and found that SO_2_ led to sulfide formation, inhibiting the reaction, while CO_2_ promoted surface carbonate formation, also unfavorable for catalysis. These findings suggest that while stronger oxidants may enhance reactivity, they often contribute to more severe catalyst deactivation.^[105]^

Since studies have shown that coke can contribute to the creation of additional active sites in the conversion of ethylbenzene to styrene, research is needed to balance the strong and moderately acidic sites to reduce the negative impact of coke and enhance its positive role.

Scalability is further constrained by infrastructure limitations, particularly the lack of cost‐effective technologies for N₂O capture, transportation, and storage. Research is underway to capture N_2_O from industrial and agricultural sources to reduce the amount of N_2_O emitted to the atmosphere.^[106]^ Based on these studies, optimized N_2_O capture technologies for each emission profile should be supported. Furthermore, economic technologies for transporting and storing N_2_O should be developed to make N_2_O introduction into processes competitive.

In conclusion, N₂O‐assisted selective oxidation processes present a promising approach to converting greenhouse gases into valuable chemicals while addressing environmental concerns. Continued innovation in catalyst design, process optimization, and infrastructure development will be critical to overcoming existing barriers and unlocking the full potential of this technology for sustainable chemical production.

## Conflict of Interests

The authors declare no conflict of interest.

## Biographical Information

*Seonho Lee earned a B.S. in Environmental Safety Engineering from Ajou University in 2022 and an M.S. in the Department of Global Smart City at Sungkyunkwan University (SKKU), South Korea.Since 2021, she has been conducting research on renewable resource production from waste as a member of the Eco‐friendly Carbon Utilization Laboratory. Currently, she is pursuing a Ph.D. in the Department of Global Smart City at SKKU, where she focuses on innovative waste valorization strategies. Her research interests includes waste‐to‐resource conversion, thermal catalytic processes, chemical recycling, sustainable process development, and functional material synthesis from waste*.

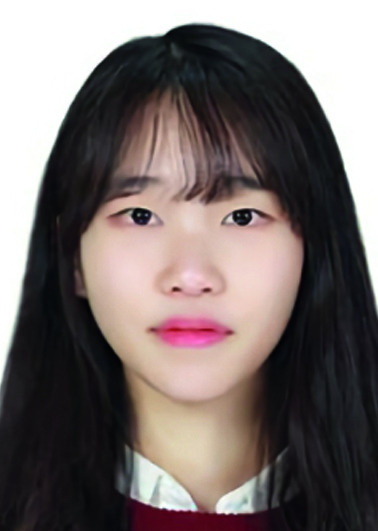

## Biographical Information

*Prof. Seong‐Jik Park received his B.S. degree from Seoul National University in 2004 and earned his Ph.D. from the same institution in 2010. After completing his doctoral studies, he worked as a postdoctoral researcher at KAIST until February 2012. In 2012, he was appointed as the youngest professor at Hankyong National University, where he currently serves as a full professor. To date, Professor Park has published 155 SCI papers and 64 articles in domestic journals. Under his supervision, 4 Ph.D. and 31 M.S. degrees have been awarded. His research interest includes greenhouse gases monitoring and reduction, water treatment, sediment remediation, and nutrient management*.

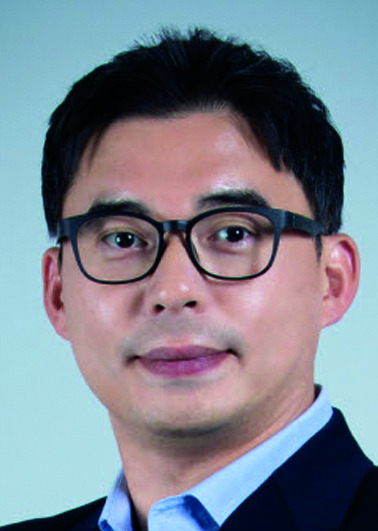

## Biographical Information

*Prof. Jechan Lee received MS in environmental engineering from Columbia University in 2010 and Ph.D. in chemical engineering from University of Wisconsin–Madison in 2015. After his PhD, he worked as a postdoctoral researcher at University of Delaware in 2015–2016 and Sejong University in 2016–2018. He then joined as a faculty member of Ajou University in 2018. He is currently an associate professor at Sungkyunkwan University (SKKU), South Korea and serves as the leader of Eco‐friendly Carbon Utilization Laboratory (ECUL). His research interest includes sustainable materials, waste upcycling, waste‐to‐energy, biorefinery, heterogeneous catalysis, and green/sustainable chemistry. He has authored more than 280 peer‐reviewed SCI(E) journal articles. He has been continuously awarded as a Highly Cited Researcher (HCR) by Clarivate Analytics since 2022. He is currently serving as an associate editor of ‘Energy & Environment’ and ‘Korean Journal of Chemical Engineering’*.